# Astrocytic YAP protects the optic nerve and retina in an experimental autoimmune encephalomyelitis model through TGF-β signaling

**DOI:** 10.7150/thno.60031

**Published:** 2021-07-25

**Authors:** Qian Wu, Xuemeng Miao, Jingjing Zhang, Ludan Xiang, Xiuchun Li, Xiaomei Bao, Siyu Du, Mianxian Wang, Shuangda Miao, Yiren Fan, Wei Wang, Xingxing Xu, Xiya Shen, Danlu Yang, Xiwu Wang, Yuanyuan Fang, Lixin Hu, Xuyi Pan, Haoru Dong, Hui Wang, Ying Wang, Jia Li, Zhihui Huang

**Affiliations:** 1 School of pharmacy and Holistic Integrative Pharmacy Institutes, Hangzhou Normal University, Hangzhou, Zhejiang, 311121, China.; 2 School of Mental Health, Wenzhou Medical University, Wenzhou, Zhejiang, 325035, China.; 3 Phase I Clinical Research Center, Zhejiang Provincial People's Hospital of Hangzhou Medical College, Hangzhou, Zhejiang, 310053, China.; 4 The First Affiliated Hospital of Wenzhou Medical University, Wenzhou, Zhejiang, 325035, China.; 5 School of Basic Medical Sciences, Wenzhou Medical University, Wenzhou, Zhejiang, 325035, China.

**Keywords:** optic neuritis, astrocytes, YAP, TGF-β1, neuroinflammation

## Abstract

**Rationale:** Optic neuritis is one of main symptoms in multiple sclerosis (MS) that causes visual disability. Astrocytes are pivotal regulators of neuroinflammation in MS, and astrocytic yes-associated protein (YAP) plays a critical role in neuroinflammation. Meanwhile, YAP signaling is involved in visual impairment, including glaucoma, retinal choroidal atrophy and retinal detachment. However, the roles and underlying mechanisms of astrocytic YAP in neuroinflammation and demyelination of MS-related optic neuritis (MS-ON) remains unclear.

**Methods:** To assess the functions of YAP in MS-ON, experimental autoimmune encephalomyelitis (EAE, a common model of MS) was established, and mice that conditional knockout (CKO) of YAP in astrocytes, YAP^GFAP^-CKO mice, were successfully generated. Behavior tests, immunostaining, Nissl staining, Hematoxylin-Eosin (HE) staining, TUNEL staining, Luxol Fast Blue (LFB) staining, electron microscopy (EM), quantitative real-time PCR (qPCR), gene set enrichment analysis (GSEA) and gene set variation analysis (GSVA) by RNA sequencing were used to examine the function and mechanism of YAP signaling based on these YAP^GFAP^-CKO mice and EAE model mice. To further explore the potential treatment of YAP signaling in EAE, EAE mice were treated with various drugs, including SRI-011381 that is an agonist of transforming growth factor-β (TGF-β) pathway, and XMU-MP-1 which inhibits Hippo kinase MST1/2 to activate YAP.

**Results:** We found that YAP was significantly upregulated and activated in the astrocytes of optic nerve in EAE mice. Conditional knockout of YAP in astrocytes caused more severe inflammatory infiltration and demyelination in optic nerve, and damage of retinal ganglion cells (RGCs) in EAE mice. Moreover, YAP deletion in astrocytes promoted the activation of astrocytes and microglia, but inhibited the proliferation of astrocytes of optic nerve in EAE mice. Mechanically, TGF-β signaling pathway was significantly down-regulated after YAP deletion in astrocytes. Additionally, both qPCR and immunofluorescence assays confirmed the reduction of TGF-β signaling pathway in YAP^GFAP^-CKO EAE mice. Interestingly, SRI-011381 partially rescued the deficits in optic nerve and retina of YAP^GFAP^-CKO EAE mice. Finally, activation of YAP signaling by XMU-MP-1 relieved the neuroinflammation and demyelination in optic nerve of EAE mice.

**Conclusions:** These results suggest astrocytic YAP may prevent the neuroinflammatory infiltration and demyelination through upregulation of TGF-β signaling and provide targets for the development of therapeutic strategies tailored for MS-ON.

## Introduction

Multiple sclerosis (MS) is the most common demyelinating disease of the central nervous system (CNS) 1, and optic neuritis, one of the main symptoms of MS 2, forces 30% of MS patients to seek medical treatments in the initial course and affects 70% of ones in the later course 3. It manifests as acute reductions of visual field size, color vision, central visual acuity, and afferent pupillary function 4, which impacts the quality of life seriously 5. But until now, there has been no effective treatment for permanent vision loss or trauma caused in MS-related optic neuritis (MS-ON) 6.

MS-ON, a kind of immune inflammatory disorder, can lead to neuroinflammation and demyelination of optic nerve, axonal damage and loss, and retinal ganglion cells (RGCs) death. Experimental autoimmune encephalomyelitis (EAE) has been used as a common model of MS to study the mechanism of MS-ON 4, 7. Nowadays, several studies have shown that astrocytes play important roles in immune inflammatory disorders 8. For example, inhibition of astrocytic NF-κB has recently been reported to improve functional outcome in EAE mice 9. The importance of astrocytes in immune inflammatory disorders is further supported by recent clinical evidence that the majority of patients with neuromyelitis optics, a CNS inflammatory disease with similarities to MS, have autoantibodies to aquaporin-4, a molecule expressed in CNS only on astrocyte foot processes 10, and that the severity of clinical signs in such patients correlates with aquaporin-4-specific antibody titers 11. Furthermore, previous studies have shown that a loss or disruption of barrier functions of scar-forming reactive astrocytes may represent a pathophysiological mechanism that could precipitate or exacerbate CNS pathology in acquired immune inflammatory disorders of the CNS, including MS or related conditions 12, 13. Functionally, astrocytes are activated and proliferate, which contributes to regulating demyelination of the axons and plays a significant role in neuroinflammation of EAE 14. However, the detailed roles and underlying mechanisms of astrocytes in the neuroinflammation and demyelination of MS-ON remains unclear.

Yes-associated protein (YAP) and its paralog, transcriptional coactivators with PDZ-binding motif (TAZ), which is also known as WW Domain Containing Transcription Regulator 1 (WWTR1), control cell proliferation, tissue homeostasis, and organ size 15, 16, and act as transcriptional co-activators that are known to be downstream effectors of the conserved Hippo kinase pathway 17, 18. Previous studies have reported that YAP plays critical roles in regulating proliferation and differentiation of astrocytes 19-21 and has a tremendous impact on Schwann cell myelination 22 and neuroinflammation 23. Additionally, YAP signaling is involved in vision-related diseases such as glaucoma, retinal choroidal atrophy and retinal detachment 24-26. However, it remains unknown whether YAP participates in MS-ON and its functions on the activated astrocytes, demyelination and neuroinflammation.

Additionally, transforming growth factor-β (TGF-β) is a multifunctional regulator of cell activity, which plays an important role in regulating the function of inflammatory immune cells and myelination 27-29. Some studies have shown that the expression of TGF-β was significantly elevated in EAE mice 30. In EAE mice model, overexpression of TGF-β could attenuate the incidence and severity of EAE notably 31-33. However, the role of TGF-β is cell type-dependent, and the combined effect of different factors makes the biological effects of TGF-β more complicated 34, 35. Recent studies have shown that YAP expression was elevated in HSCT6 cells treated with TGF-β, and promoted the activation and proliferation of HSCT6 cells 36. TGF-β can restrain nuclear translocation of YAP/TAZ at the mRNA and protein levels 37, while YAP can regulate the cell response of TGF-β effectively 38. However, the interaction between YAP and TGF-β in MS-ON remains unclear.

Results of the present study demonstrated that YAP in astrocytes was activated and upregulated in optic nerve of EAE mice, and conditional ablation of YAP in astrocytes aggravated inflammatory infiltration and demyelination, and resulted into more astrocytic and microglial activation in optic nerve and retina of EAE mice, associated with a drastic increasing of RGCs loss in retina of EAE mice. Mechanistically, YAP signaling prevented demyelination and inflammatory response through TGF-β pathways. Our study reveals the underlying mechanisms of demyelination and inflammation controlled by astrocytes in optic neuritis, which may help to develop new strategies for the treatment of MS-ON.

## Material and Methods

### Mouse breeding and genotyping

YAP^GFAP^-CKO mice were generated by crossing floxed YAP allele (YAP^f/f^) with GFAP-Cre transgenic mice. The origin of our GFAP-Cre mice is GFAP-Cre 8Gtm (from Jackson Labs), and YAP^f/f^ mice were generated as previously described 39. YAP^f/f^ and YAP^GFAP^-CKO mice were maintained in the background of C57BL/6 strain and identified by genotyping. All mice were divided into experimental groups randomly. Additionally, genotyping and experimental condition sets were performed blindly. Moreover, the histological and behavioral tests were conducted with strict compliance to the relevant regulations of Animal Bioethics Committee of Hangzhou Normal University and Wenzhou Medical University.

### Establishment of EAE model

EAE model was performed as described previously 40. Briefly, female C57BL/6 mice (8-10 weeks) were anesthetized and then subcutaneously immunized with 0.2 mg of myelin oligodendrocyte glycoprotein 35-55 (MOG_35-55_) [GL Biochem (Shanghai) Ltd] emulsified (1:1) in Freund's complete adjuvant containing 8 mg/ml mycobacterium tuberculosis (strain H37RA, Difco, Detroit, Michigan, USA). Mice were divided into 2 groups: the mice in control group received subcutaneous injection of phosphate-buffered saline (PBS), while the mice in the model experiment (EAE) group were injected subcutaneously with the MOG_35-55_/Freund's complete adjuvant emulsion in PBS. Furthermore, the mice were intraperitoneally injected with 300 ng of pertussis toxin (Sigma, USA) on day 0 and 2 after immunization. All injection and scoring processes were double-blinded. From 0 to 22 days post injection (dpi), body weight was recorded and the neurological function was assessed according to five-point scoring criteria published previously (All animals were examined and scored daily for clinical signs of EAE). Animals were clinically graded as follows: 0 = no signs, 1 = loss of tail tonicity, 2 = loss of tail tonicity and mild paralysis of hindlimbs, 3 = paralysis of hindlimbs, 4 = hindlimb paralysis and mild paralysis of forelimbs and 5 = complete paralysis or death. All mice were sacrificed at 22 dpi 41.

### Injection of drugs and reagents

After EAE induction, SRI-011381 (HY-100347A, MCE), dissolved in saline containing DMSO (10%) and PEG300 (40%), was injected intraperitoneally into mice at a dose of 30 mg/kg every 2 d 42. XMU-MP-1 (HY-100526, MCE) was dissolved in DMSO and injected intraperitoneally at a dose of 1 mg/kg, given every 2 d 43. At the end of the study, the optic nerve and retina were collected for further investigations.

### Hematoxylin-Eosin (HE) staining

Briefly, after perfusion with PBS and then with 4% paraformaldehyde (PFA), the optic nerve and retina tissues were immersed in 4% PFA for 24 h and transferred to 30% sucrose solution until they sank. Subsequently, the optic nerve and retina tissues were embedded in optimal cutting temperature and a cryostat (Thermo, USA) was used to cut into a 5 μm-thick longitudinal section of optic nerve and a 10 μm-thick cross section of retina. After dyeing with hematoxylin for 1.5 min, the sections were rinsed with double distilled water for 3 times. Then the sections were incubated in acidic liquid alcohol for 30 s, stained with eosin for 50 s, then rinsed with 95% ethanol and dehydrated with 100% ethanol, finally removed with xylene and fixed with neutral resin. Additionally, the sections were observed under Olympus SLIDEVIEW^TM^ VS200 microscope (Olympus, Germany). The quantitative analysis of the images was made by Image J.

### Nissl staining

Nissl staining was conducted as described previously 43. Briefly, 10 μm-thick retina tissues were incubated with 0.1% cresyl violet for 6 min at room temperature, slices were rinsed with double distilled water, then dehydrated in 95% and 100% ethanol in succession, clarified with xylene, and ultimately fixed with neutral resin. The sections were observed under Olympus SLIDEVIEW^TM^ VS200 microscope (Olympus, Germany). The quantitative analysis of the image was made by Image J.

### TUNEL staining

TUNEL staining of 10 μm-thick retina tissues was conducted to evaluate apoptosis as described previously 44. After washing with PBS, the tissues were blocked in 5% BSA plus 0.3% Triton X-100 for 1 h at room temperature, and treated with *in situ* detection kit according to the manufacturer's protocol (MK500, TaKaRa Bio Inc, Shiga, Japan) at room temperature for 1.5 h. The sections were then observed under Olympus SLIDEVIEW^TM^ VS200 microscope (Olympus, Germany) and processed by Image J or Adobe Photoshop CC 14.0 software.

### Luxol Fast Blue (LFB) staining

LFB staining was conducted as described previously 45. Briefly, 5 μm-thick optic nerve tissues were stained in LFB staining solution overnight at room temperature. Following the staining, the slices were rinsed in 95% ethanol, washed with distilled water, rinsed in 70% ethanol, and later with distilled water. Next, the slices were counterstained in cresyl violet working solution for 15 s, and then dehydrated in 95% and 100% ethanol in succession, differentiated in xylene until there was no background color. Finally, slices were sealed with neutral resin. The sections were then observed under Olympus SLIDEVIEW^TM^ VS200 microscope (Olympus, Germany). The quantitative analysis of the images was performed by image J.

### Electron Microscopy (EM)

For EM, optic nerve tissues were fixed overnight by immersion in 2.5% glutaraldehyde. Then samples were postfixed with 1% osmium and 1% uranyl acetate for 1 h respectively, dehydrated in ethanol, and embedded in resin. The samples were cut into ultrathin sections to receive staining with uranyl acetate. Finally, the sections were used for capturing images with a Hitachi H-600 EM (HITACH, Tokyo, Japan).

### Cell culture

Primary astrocyte cultures were prepared from the cerebral cortex of P1-P3 YAP^f/f^ and YAP^GFAP^-CKO mice as described previously 39. In brief, cerebral neocortex was dissected, chopped and then incubated with 0.125% trypsin (Gibco) at 37 ℃ for 15 min, and separated into single cell suspension by mechanical destruction. The cells were seeded on culture flasks coated with poly-L-lysine (0.1 mg/ml, Sigma) and cultured with DMEM containing 10% fetal bovine serum (FBS, Gibco). After 6-10 d, the microglia and oligodendrocytes were removed by shaking at 250 rpm for about 5 h. Then astrocytes were isolated and plated into a poly-D-lysine coated Petri dish or cover glass. The purity of GFAP^ +^ cells was at least 94%.

### Immunostaining

Immunostaining of cultured astrocytes or 5 μm-thick optic nerve tissues or 10 μm-thick retina tissues were conducted as previously described 46. For cultured cells, briefly, cells were received 3 times of rinses with PBS, and then fixed in 4% PFA for 15 min. Next, after being blocked and permeabilized with 0.1% Triton X-100 in PBS containing 5% bovine serum albumin (BSA) at room temperature for 45 min, cells were incubated with primary antibodies at 4 ℃ for overnight, rinsing in PBS for 3 times and another incubation with secondary antibodies (Invitrogen, 1:1, 000) at room temperature for 1 h. Primary antibodies included rabbit anti-YAP (ab205270, Abcam, 1:200) and mouse anti-GFAP (MAB360, Millipore, 1:500). After rinsing in PBS for another 3 times, cells were mounted.

The tissues were fixed with fresh 4% PFA after cardiac perfusion for 1 d. All tissues were dehydrated in PBS with 30% sucrose until they sank, and sliced on frozen microtome. After washing for 3 times, 5 μm-thick longitudinal sections of optic nerve and 10 μm-thick cross sections of retina were then blocked at room temperature for 1 h in 5% BSA plus 0.3% Triton X-100, incubated with the primary antibody overnight at 4 ℃, rinsed with PBS for 3 times and incubated with the corresponding secondary antibodies (Invitrogen, 1:1, 000) at room temperature for 1 h. The primary antibodies included mouse anti-YAP (WH0010413M1, Sigma, 1:200), rabbit anti-YAP (ab205270, Abcam, 1:500), rabbit anti-GFAP (ab68428, Abcam, 1:250), mouse anti-GFAP (MAB360, Millipore, 1:500), goat anti-Iba1 (ab5076, Abcam, 1:500), rabbit anti-Ki67 (AB9260, Millipore, 1:300), mouse anti-PH3 (ab14955, Abcam, 1:500), rabbit anti-CD45 (ab10558, Abcam, 1:500), rabbit anti-CD206 (ab125028, Abcam, 1:200), mouse anti-MBP (ab62631, Abcam, 1:500), rabbit anti-RBPMS (GTX118619, GeneTex, 1 : 500), rabbit anti-NeuN (ab177487, Abcam, 1:200), mouse anti-NeuN (ab104224, Abcam, 1:200), rabbit anti-TGF-β1 (ab215715, Abcam, 1:200) and rabbit anti-Vimentin (ab92547, Abcam, 1:800). Sections were stained for DAPI (Sigma, 1:1, 000) to visualize nucleus. Images were visualized by Olympus SLIDEVIEW^TM^ VS200 microscope (Olympus, Germany) and processed by Image J or Adobe Photoshop CC 14.0 software. No positive signal was observed in the control group.

### Immunohistochemistry

The antigens were prepared using Sodium Citrate Antigen Retrieval Solution for 30 min at 90 ℃. Subsequently, the endogenous peroxidase activity of sections was blocked with 3% H_2_O_2_ for 0.5 h and 5% BSA plus 0.3% Triton X-100 at room temperature for 1 h. Following blocking, the sections were incubated with rabbit anti-YAP antibody (ab205270, Abcam, 1:200) overnight at 4 ℃, and then washed 3 times with PBS and incubated for 1 h with HRP-conjugated goat anti-rabbit IgG (Abcam, 1:100). Immunoreactivity was detected by using diaminobenzidine. All antibodies used for immunohistochemistry were diluted in PBS containing 5% BSA. The sections were then observed under Olympus SLIDEVIEW^TM^ VS200 microscope (Olympus, Germany). The brown yellow particles in the nucleus were judged as YAP positive (YAP^+^) cells. No positive signal was observed in the control group.

### RNA sequencing and functional enrichment analysis

Total RNA was extracted from cultured YAP^+/+^ and YAP^-/-^ astrocytes as well as optic nerve of control and EAE mice using Trizol reagent (Invitrogen), according to the manufacturer's protocol. The RNA samples used for sequencing were with A260:A280 ratio above 1.8, A260:A230 ratio above 2.0 and the RNA integrity number above 7.0. Briefly, rRNAs were removed from total RNA using Ribo-Zero rRNA Removal Kit (Epicentre, USA) and fragmented to approximately 200 bp. Subsequently, the purified RNAs were subjected to first strand and second strand cDNA synthesis following by adaptor ligation and enrichment with a low-cycle according to instructions of NEBNext® Ultra™ RNA Library Prep Kit for Illumina (NEB, USA). The purified library products were evaluated using the Agilent 2200 Tape Station and Qubit®2.0 (Life Technologies, USA) and then diluted to 10 pM for cluster generation *in situ* on the pair-end flow cell followed by HiSeq3000sequencing (2×150 bp). The clean reads were obtained after removal of reads containing adapter, ploy-N and at low quality from raw data. HISAT2 was used to align the clean reads to the mouse reference genome mm10 with default parameters. HTSeq was subsequently employed to convert aligned short reads into read counts for each gene model. Differential expression was assessed by DEseq using read counts as input. The Benjamini-Hochberg multiple test correction method was enabled. Gene set enrichment analysis (GSEA) was performed with all expressed genes using GSEA software (version 4.0.2). Gene sets with normal p-value < 0.05 and FDR < 0.25 were considered significantly enriched. These gene sets were visualized by “ggplot2” (R package). In addition, and “gene set variation analysis (GSVA)” (R package) was utilized to perform GSVA to validate the gene sets of GSEA. The gene set “hall.v7.0.symbols.gmt” was selected as the reference gene set. P-value < 0.05 was considered as statistically significant. Ultimately, “p-heatmap” (R package) was utilized to show the enrichment of all gene sets in each group and the gene expression in the selected gene set.

### RNA extraction and quantitative real-time PCR (qPCR)

Total RNA from cells was extracted by using TRIzol^TM^ reagent (#15596026, Ambion) to determine the mRNA expression levels of genes according to protocol provided by the manufacturer. The total RNA (2 μg) was reversely transcribed into cDNA with a SuperScript™ One-Step Reverse Transcription Kit (#10928-034, Invitrogen). The expression levels of TGF-β1, Smad1, Smad2, Smad3, Smad4, Ccl8, Ccl9, IL-1β and TNF-α mRNA were quantified by iTaq™ Universal SYBR® Green Supermax (#172-5122, Bio-Rad) on Real-Time PCR detection System (Applied Biosystems, USA), which using β-actin as the endogenous control. The relative levels of mRNA expression were represented as ΔCt = Ct gene-Ct reference, and the multiple change of gene expression was calculated by the 2-ΔΔCt method. The primers used in this research were synthesized by Sang on Biotech, presented below:

TGF-β1 primer: forward, 5'-TGGAGCAACATGTGGAACTC-3' and reverse, 5'-GTCAGCAGCCGGTTACCA-3'; Smad1 primer: forward, 5'-GCGGGTGCAGCTTGAAAATC-3' and reverse, 5'-GCACTGCTCACCTTCACGA-3'; Smad2 primer: forward, 5'-AAGCCATCACCACTCAGAATTG-3' and reverse, 5'-CACTGATCTACCGTATTTGCTGT-3'; Smad3 primer: forward, 5'-CACGCAGAACGTGAACACC-3', and reverse, 5'-GGCAGTAGATAACGTGAGGGA-3'; Smad4 primer: forward, 5'-ACACCAACAAGTAACGATGCC-3', and reverse, 5'-GCAAAGGTTTCACTTTCCCCA-3'; Ccl8 primer: forward, 5'-TTCTTTGCCTGCTGCTCATA-3', and reverse, 5'-GCAGGTGACTGGAGCCTTAT-3'; Cc19 primer: forward, 5'-TGGGCCCAGATCACACAT-3', and reverse, 5'-TGTGAAACATTTCAATTTCAAGC-3'; IL-1β primer: forward, 5'-GTGTGGATCCAAAGCAATAC-3', and reverse, 5'-GTCTGCTCATTCATGACAAG-3'; TNF-α primer: forward, 5'-GGGGCCACCACGCTCTTCTGTC-3', and reverse, 5'-TGGGCTACGGGCTTGTCACTCG-3'; β-actin primer: forward, 5'-AAGTCCCTCACCCTCCCAAAAG-3' and reverse 5'-AAGCAATGCTGTCACCTTCCC-3'.

### Statistical Analysis

All results were expressed as mean ± SEM derived from at least 3 independent experiments. GraphPad Prism software was used for statistical analysis. Student's t-test, or ANOVA analysis were utilized. *P < 0.05* was considered as statistically significant.

## Results

### YAP is upregulated and activated in optic nerve astrocytes of EAE mice

In order to explore potential functions of YAP in MS-ON, EAE, a common typical model of MS, was established. C57BL/6 female mice (8-10 weeks) were immunized with MOG_35-55_ and a pertussis toxin boost. As expected, these mice were developed an acute monophasic EAE, and at 22 dpi, the optic nerve and retina of EAE mice were collected. Then LFB staining and myelin basic protein (MBP) (a marker of myelin) immunostaining showed obvious demyelination in optic nerve of EAE mice (Supplementary Figure S1A-D). Furthermore, immunostaining of CD45 (a marker of T cells) and GFAP (a marker of astrocytes) also showed the obvious inflammatory infiltration in optic nerve of EAE mice (Supplementary Figure S1E-H). These results indeed indicated that optic neuritis of EAE model was established successfully.

**Figure 1:**
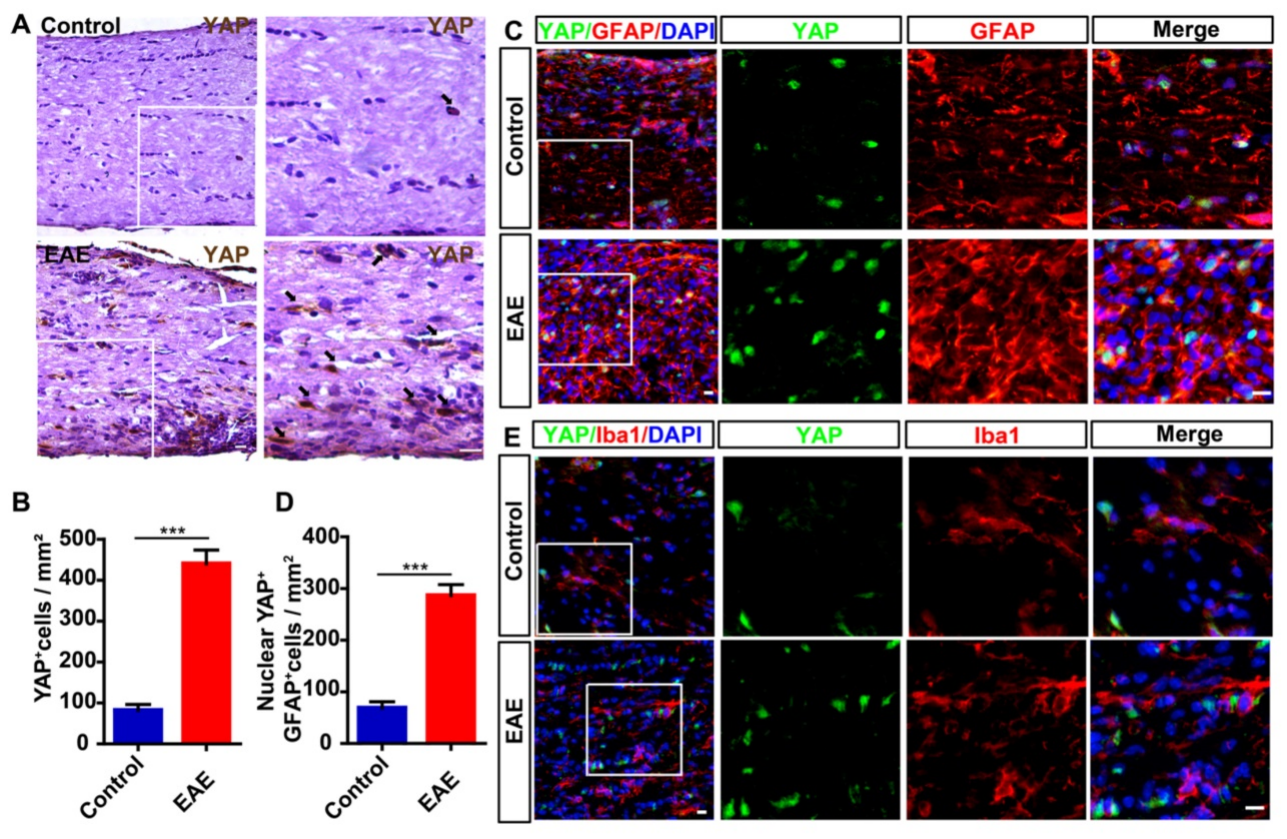
** YAP is upregulated and activated in optic nerve astrocytes of EAE mice.** (A) Immunohistochemistry detected YAP expression in the optic nerve of control and EAE mice. (B) Quantitative analysis of the density of YAP^+^ cells as shown in (A) (n = 8 per group). (C) Double immunostaining of YAP (green) and GFAP (red) in the optic nerve of control and EAE mice. (D) Quantitative analysis of the density of nuclear YAP^+^ astrocytes as shown in (C) (n = 6 per group). (E) Double immunostaining of YAP (green) and Iba1 (red) in the optic nerve of control and EAE mice. Images of selected regions (white squares) were shown at higher magnification. Data were mean ± SEM, Student's t-test, compared with control group, ^***^P < 0.001. Scale bars, 20 μm.

Next, we examined the expression level of YAP in optic nerve of EAE mice. As shown in Figure 1A-B, compared with control mice, the expression level of YAP was significantly increased in optic nerve of EAE mice. To further examine the cellular expression pattern of YAP in optic nerve, double-immunostaining of YAP and several cell markers including GFAP or Iba1 (a marker of microglia) was performed in optic nerve of control mice and EAE mice, respectively, and showed that YAP was mainly expressed in GFAP^+^ astrocytes (Figure 1C), but not in Iba1^+^ microglia (Figure 1E), and YAP was upregulated and displayed the nuclear location in GFAP^+^ astrocytes of EAE optic nerve (Figure 1C-D). Taken together, these results suggest that YAP is upregulated and activated in optic nerve astrocytes of EAE mice.

### The normal development of optic nerve and retina in YAP^GFAP^-CKO mice

To further investigate the role of astrocytic YAP in MS-ON, conditional YAP knockout mice in astrocytes were generated through crossing YAP^f/f^ with GFAP-Cre transgenic mice, namely YAP^GFAP^-CKO mice (Supplementary Figure S2A-B). Indeed, YAP was knockout in GFAP^+^ astrocytes *in vitro* and* in vivo* (Supplementary Figure S2C-D). However, there was no significant difference in body weight between YAP^f/f^ and YAP^GFAP^-CKO mice (Supplementary Figure S2E). To further examine whether YAP deletion in astrocytes affected the development of visual system, HE staining was performed and showed that conditional knockout YAP in astrocytes did not significantly affect the morphology of optic nerve and retina (Supplementary Figure S3A-B). Moreover, immunostaining of GFAP and Iba1 showed that the distribution and number of astrocytes and microglia were not affected in optic nerve by YAP knockout (Supplementary Figure S3C-D). Consistent with the results shown in optic nerve, immunostaining of GFAP, Iba1 and RBPMS (a marker of RGCs) in retina also showed that there were no significant differences in the distribution and number of astrocytes, microglia and RGCs between YAP^f/f^ and YAP^GFAP^-CKO mice (Supplementary Figure S3E-G). Taken together, these results suggest that YAP deletion in astrocytes does not affect the normal development of optic nerve and retina.

### Conditional knockout of YAP in astrocytes exacerbates the demyelination in optic nerve of EAE mice

**Figure 2:**
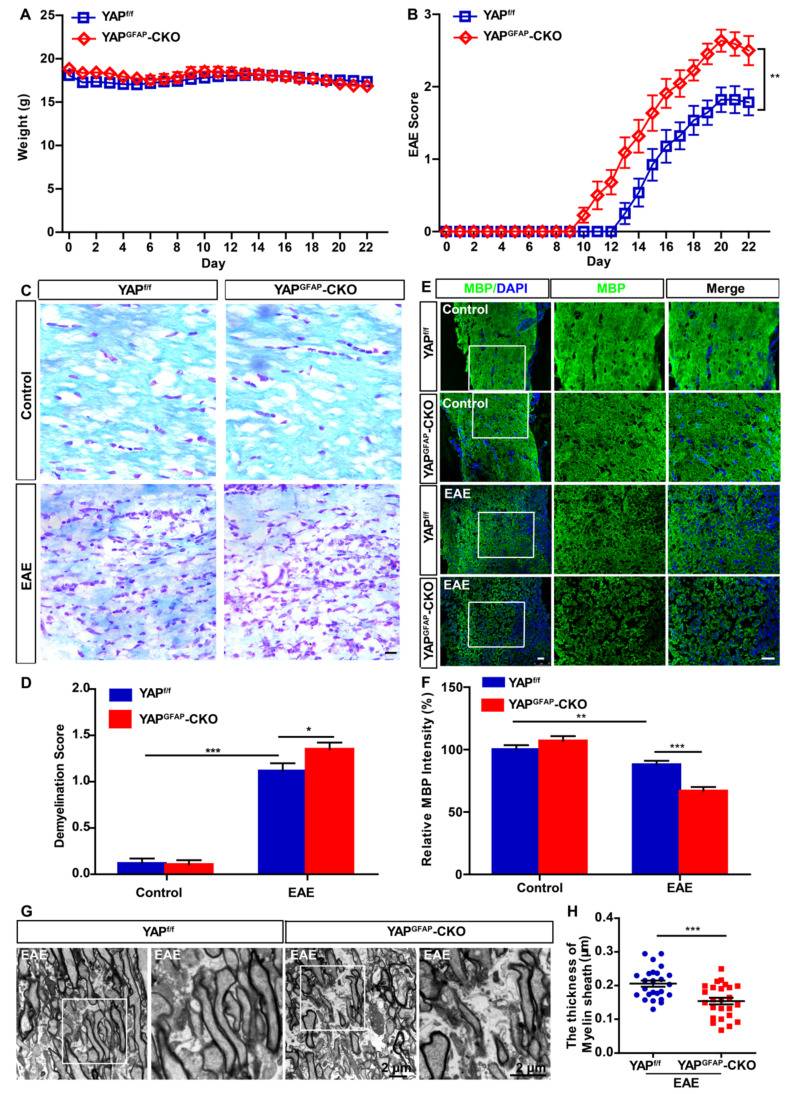
** Ablation of YAP in astrocytes exacerbates the demyelination in optic nerve of EAE mice.** (A) The body weight of YAP^f/f^ (n = 14) and YAP^GFAP^-CKO (n = 11) mice from 0 to 22 dpi during the process of EAE modeling (two-way ANOVA with Bonferroni's post-tests). (B) The EAE score of YAP^f/f^ (n = 14) and YAP^GFAP^-CKO (n = 11) mice 0 to 22 dpi during the process of EAE modeling (two-way ANOVA with Bonferroni's post-tests). (C) LFB staining of optic nerve obtained from YAP^f/f^ and YAP^GFAP^-CKO mice, YAP^f/f^ EAE and YAP^GFAP^-CKO EAE mice. (D) Quantitative analysis of the demyelination score as shown in (C) (n = 12 per group, two-way ANOVA with Bonferroni's post-tests). (E) Immunostaining of MBP (green) in the optic nerve obtained from YAP^f/f^ and YAP^GFAP^-CKO mice, YAP^f/f^ EAE and YAP^GFAP^-CKO EAE mice. (F) Quantitative analysis of MBP intensity as shown in (E) (n = 12 per group, two-way ANOVA with Bonferroni's post-tests). (G) Transmission EM images of the longitudinal section of the optic nerve obtained from YAP^f/f^ EAE and YAP^GFAP^-CKO EAE mice. (H) Quantitative analysis of the thickness of myelin sheath as shown in (G) (n = 25 per group, Student's t-test). Images of selected regions (white squares) were shown at higher magnification. Data were mean ± SEM, compared with YAP^f/f^ group.^ *^P < 0.05, ^**^P < 0.01, ^***^P < 0.001. Scale bars, 20 μm.

YAP^f/f^ and YAP^GFAP^-CKO mice were next injected with MOG_35-55_ and pertussis toxin to induce EAE, as shown in Figure 2A, there was no significant difference in body weight between YAP^f/f^ EAE and YAP^GFAP^-CKO EAE mice. However, deletion of YAP in astrocytes resulted in worse clinical scores. The first clinical signs (tip of tail was limp) were advanced in YAP^GFAP^-CKO EAE mice, observed at 13-15 and 10-12 dpi, in YAP^f/f^ EAE mice and YAP^GFAP^-CKO EAE mice, respectively (Figure 2B). Additionally, since demyelination is one of the features of EAE 47, we next examined whether conditional knockout of YAP in astrocytes would affect the demyelination in optic nerve of EAE mice. As expected, LFB staining and MBP immunostaining in optic nerve indeed showed that there was a significant increase in demyelination score and decrease in MBP intensity of YAP^GFAP^-CKO EAE mice (Figure 2C-F). Furthermore, EM experiment further showed that YAP^GFAP^-CKO EAE mice had more severe demyelination phenotype in optic nerve than that in YAP^f/f^ EAE mice (Figure 2G-H). Taken together, our results indicate that conditional knockout of YAP in astrocytes exacerbates the demyelination in optic nerve of EAE mice.

### Conditional knockout of YAP in astrocytes exacerbates the inflammatory infiltration in optic nerve of EAE mice

**Figure 3:**
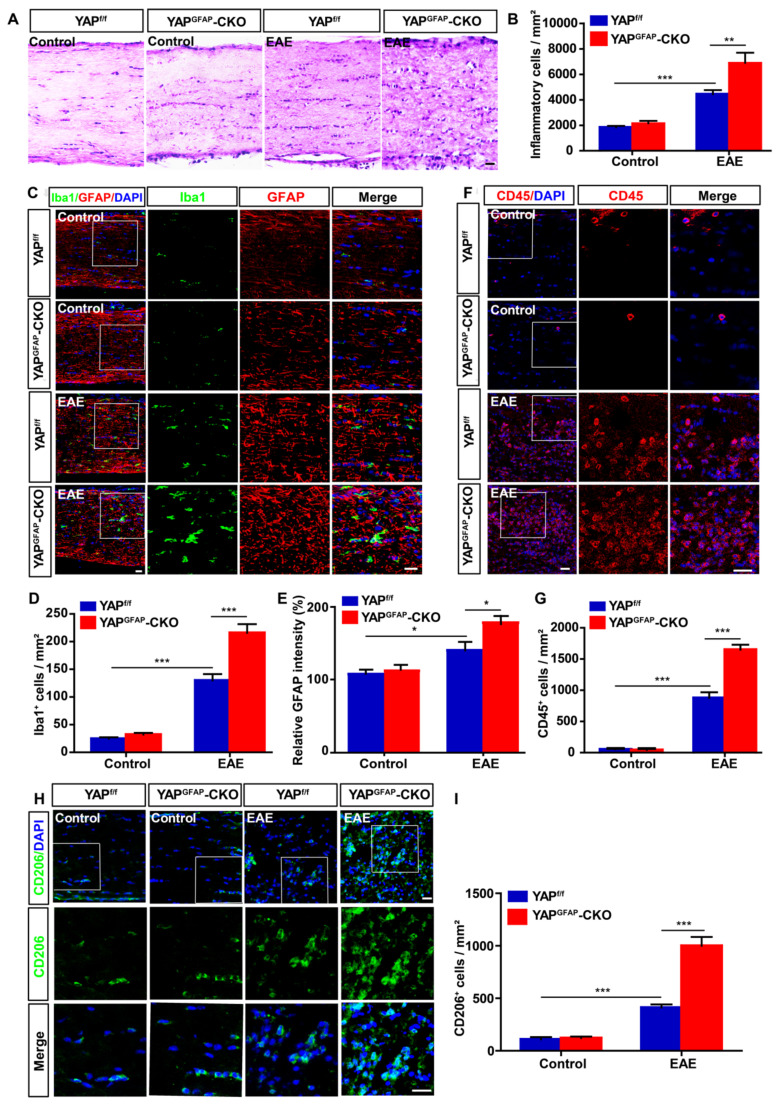
** Ablation of YAP in astrocytes exacerbates the inflammatory infiltration in optic nerve of EAE mice.** (A) HE staining of optic nerve obtained from YAP^f/f^ and YAP^GFAP^-CKO mice, YAP^f/f^ EAE and YAP^GFAP^-CKO EAE mice. (B) Quantitative analysis of the density of inflammatory cells as shown in (A) (n = 10 per group). (C) Double immunostaining of Iba1 (green) and GFAP (red) in the optic nerve obtained from YAP^f/f^ and YAP^GFAP^-CKO mice, YAP^f/f^ EAE and YAP^GFAP^-CKO EAE mice. (D) Quantitative analysis of the density of Iba1^+^ cells as shown in (C) (n = 12 per group). (E) Quantitative analysis of the relative GFAP intensity as shown in (C) (n = 9 per group, normalized to control). (F) Immunostaining of CD45 (red) in the optic nerve obtained from YAP^f/f^ and YAP^GFAP^-CKO mice, YAP^f/f^ EAE and YAP^GFAP^-CKO EAE mice. (G) Quantitative analysis of the density of CD45^+^ cells as shown in (F) (n = 9 per group). (H) Immunostaining of CD206 (green) in the optic nerve obtained from YAP^f/f^ and YAP^GFAP^-CKO mice, YAP^f/f^ EAE and YAP^GFAP^-CKO EAE mice. (I) Quantitative analysis of the density of CD206^+^ cells as shown in (H) (n = 10 per group). Images of selected regions (white squares) were shown at higher magnification. Data were mean ± SEM, two-way ANOVA with Bonferroni's post-tests, compared with YAP^f/f^ group, ^*^P < 0.05, ^**^P < 0.01, ^***^P < 0.001. Scale bars, 20 μm.

Recent studies have suggested that inflammatory infiltration has a tight connection with demyelination in EAE mice 48, 49. Thus, HE staining was performed to examine the inflammatory infiltration of optic nerve in EAE mice. As shown in Figure 3A-B, inflammatory infiltration in optic nerve of YAP^GFAP^-CKO EAE mice was aggravated obviously, compared with that in YAP^f/f^ EAE mice. As inflammatory cells under optic neuritis conditions, including microglial cells, astrocytes and T cells 50, 51, GFAP^+^ astrocytes and Iba1^+^ microglia displayed a hypertrophy morphology, and the density of microglia and intensity of GFAP were increased in YAP^GFAP^-CKO EAE mice, compared with that in YAP^f/f^ EAE mice (Figure 3C-E). These results indicated that astrocytes and microglia were activated in YAP^GFAP^-CKO EAE mice. CD45^+^ (a marker of T cells) cells and CD206^+^ (a marker of microglia) cells were also increased in optic nerve of YAP^GFAP^-CKO EAE mice (Figure 3F-I), suggesting that YAP deletion in astrocytes not only enhanced astrocytic and microglial activation, but also increased the number of microglia and T cells in optic nerve of EAE mice. Next, we performed qPCR experiments to explore expression patterns of chemokines and cytokines in YAP deficient astrocytes of the optic nerve. Results showed Ccl8 and Ccl9 were upregulated in the optic nerve of YAP^GFAP^-CKO EAE mice, compared with that in YAP^f/f^ EAE mice (Supplementary Figure S4A-B). Additionally, cytokines, including TNF-α and IL-1β, were also upregulated in optic nerve of YAP^GFAP^-CKO EAE mice (Supplementary Figure S4C-D). Therefore, we speculated that increase of Ccl8, Ccl9, IL-1β and TNF-α from YAP-deleted astrocytes may promote activation of microglia and neuroinflammation. Taken together, these results indicate that conditional knockout of YAP in astrocytes aggravates severe inflammatory infiltration in the optic nerve of EAE mice.

**Figure 4:**
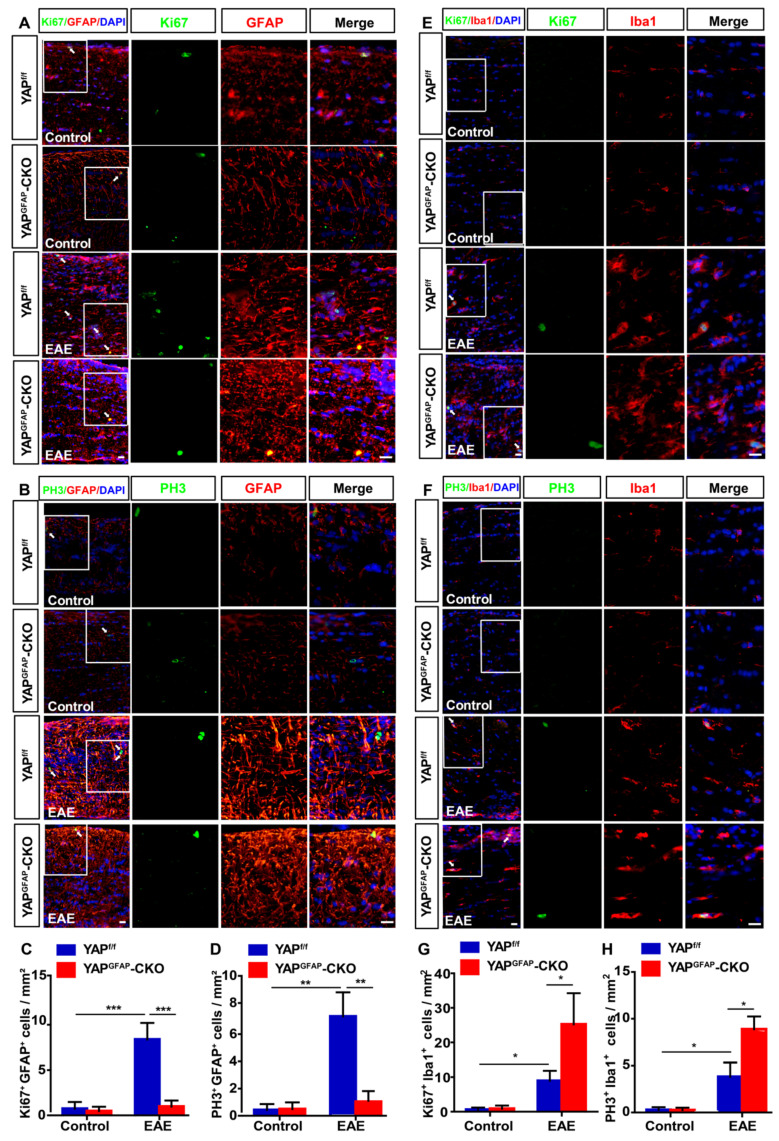
** Ablation of YAP in astrocytes inhibits the proliferation of astrocytes and promotes the proliferation of microglia in optic nerve of EAE mice.** (A-B) Double immunostaining of Ki67 (green) and GFAP (red) (A) or PH3 (green) and GFAP (red) (B) in the optic nerve of YAP^f/f^ and YAP^GFAP^-CKO mice, YAP^f/f^ EAE and YAP^GFAP^-CKO EAE mice. (C-D) Quantitative analysis of density of Ki67^+^/GFAP^+^ (C) or PH3^+^/GFAP^+^ (D) cells as shown in (A-B) (n = 7 per group). (E-F) Double immunostaining of Ki67 (green) and Iba1(red) (E), or PH3 (green) and Iba1 (red) (F) in the optic nerve of YAP^f/f^ and YAP^GFAP^-CKO mice, YAP^f/f^ EAE and YAP^GFAP^-CKO EAE mice. (G-H) Quantitative analysis of the density of Ki67^+^/Iba1^+^ (G) or PH3^+^/Iba1^+^ (H) cells as shown in (E-F) (n = 10 per group). Images of selected regions (white squares) were shown at higher magnification. Data were mean ± SEM, two-way ANOVA with Bonferroni's post-tests, compared with YAP^f/f^ group, ^*^P < 0.05, ^**^P < 0.01, ^***^P < 0.001. Scale bars, 20 μm.

Since YAP is involved in proliferation of various cells, such as astrocytes, glioma cells and olfactory ensheathing cells 39, 43, 52, to further explore whether the inflammatory infiltration in YAP^GFAP^-CKO mice was caused by the proliferation of astrocytes and microglia, double immunostaining of Ki67/GFAP, PH3/GFAP, Ki67/Iba1, and PH3/Iba1 (Ki67 and PH3, cell markers of proliferation) were performed in optic nerve of YAP^f/f^ EAE and YAP^GFAP^-CKO EAE mice. As shown in Figure 4A-D, the proliferation of astrocytes was decreased in optic nerve of YAP^GFAP^-CKO EAE mice, compared with that in YAP^f/f^ EAE mice. However, the proliferation of microglia in optic nerve of YAP^GFAP^-CKO EAE mice was increased than that in YAP^f/f^ EAE mice (Figure 4E-H), which might be due to the increase of Ccl8, Ccl9, IL-1β and TNF-α (Supplementary Figure S4A-D). These results suggest that YAP knockout in astrocytes inhibits the proliferation of astrocytes, but promotes the proliferation of microglia in optic nerve of EAE mice.

### Conditional knockout of YAP in astrocytes aggravates the inflammatory infiltration and induces more RGCs loss in retina of EAE mice

**Figure 5:**
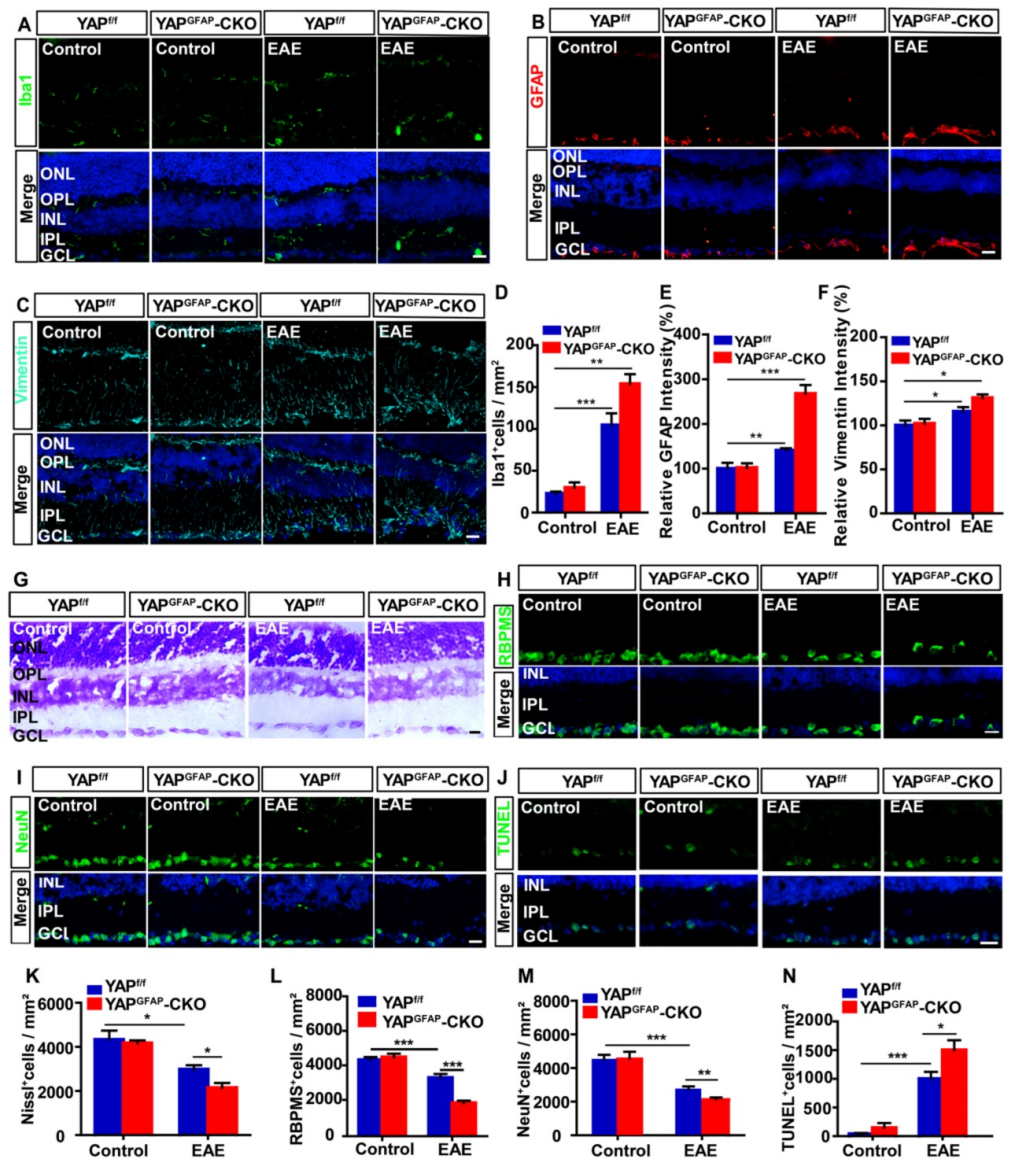
** Ablation of YAP in astrocytes exacerbates the inflammation and induces the apoptosis in retina of EAE mice.** (A-C) Immunostaining of Iba1 (green) (A), GFAP (red) (B) or Vimentin (cyan) (C) in the retina of YAP^f/f^ and YAP^GFAP^-CKO mice, YAP^f/f^ EAE and YAP^GFAP^-CKO EAE mice. (D) Quantitative analysis of the density of Iba1^+^ cells as shown in (A) (n = 7 per group). (E-F) Quantitative analysis of the relative GFAP intensity (E) or Vimentin intensity (F) as shown in (B-C) (n = 8 per group, normalized to control). (G) Representative images of Nissl staining in the retina of YAP^f/f^ and YAP^GFAP^-CKO mice, YAP^f/f^ EAE and YAP^GFAP^-CKO EAE mice. (H-I) Immunostaining of RBPMS (green) (H) or NeuN (green) (I) in the retina of YAP^f/f^ and YAP^GFAP^-CKO mice, YAP^f/f^ EAE and YAP^GFAP^-CKO EAE mice. (J) Immunostaining analysis of cell apoptosis by TUNEL staining in the retina of YAP^f/f^ and YAP^GFAP^-CKO mice, YAP^f/f^ EAE and YAP^GFAP^-CKO EAE mice. (K) Quantitative analysis of the density of Nissl^+^ cells as shown in (G) (n = 11 per group). (L-M) Quantitative analysis of the density of RBPMS^+^ cells (L) or NeuN^+^ cells (M) as shown in (H-I) (n = 7 per group). (N) Quantitative analysis of the density of TUNEL^+^ cells as shown in (J) (n = 9 per group). Data were mean ± SEM, two-way ANOVA with Bonferroni's post-tests, compared with YAP^f/f^ group, ^*^P < 0.05, ^**^P < 0.01, ^***^P < 0.001. Scale bars, 20 μm.

Inflammatory infiltration in optic neuritis is found not only in optic nerve, but also in retina 51. As expected, immunostaining showed that there was a significant increase in the density of Iba1^+^ cells and the intensity of GFAP^+^ or vimentin^+^ (a marker of reactive astrocytes) in retina of YAP^GFAP^-CKO EAE mice, compared with that in YAP^f/f^ EAE mice (Figure 5A-F). Recent studies have found that inflammatory infiltration in optic neuritis is associated with RGCs loss to some extent 53. To further test this possibility, Nissl staining showed that the density of RGCs in retina of YAP^GFAP^-CKO EAE mice was indeed significantly decreased (Figure 5G, K), compared with that in YAP^f/f^ EAE mice. Consistently, as shown in Figure 5H-I, the densities of RBPMS^+^ and NeuN^+^ cells (both RBPMS and NeuN are markers of RGCs) were also significantly decreased in retina of YAP^GFAP^-CKO EAE mice, compared with that in YAP^f/f^ EAE mice (Figure 5L-M). Furthermore, TUNEL staining showed that the apoptosis of RGCs was significantly increased in retina of YAP^GFAP^-CKO EAE mice (Figure 5J, N). Taken together, these results suggest that conditional knockout YAP in astrocytes aggravates the inflammatory infiltration in retina, which may lead to more RGCs loss in retina of EAE mice.

### Downregulation of astrocytic TGF-β signaling pathway may cause more severe neuroinflammation in optic nerve of YAP^GFAP^-CKO EAE mice

**Figure 6:**
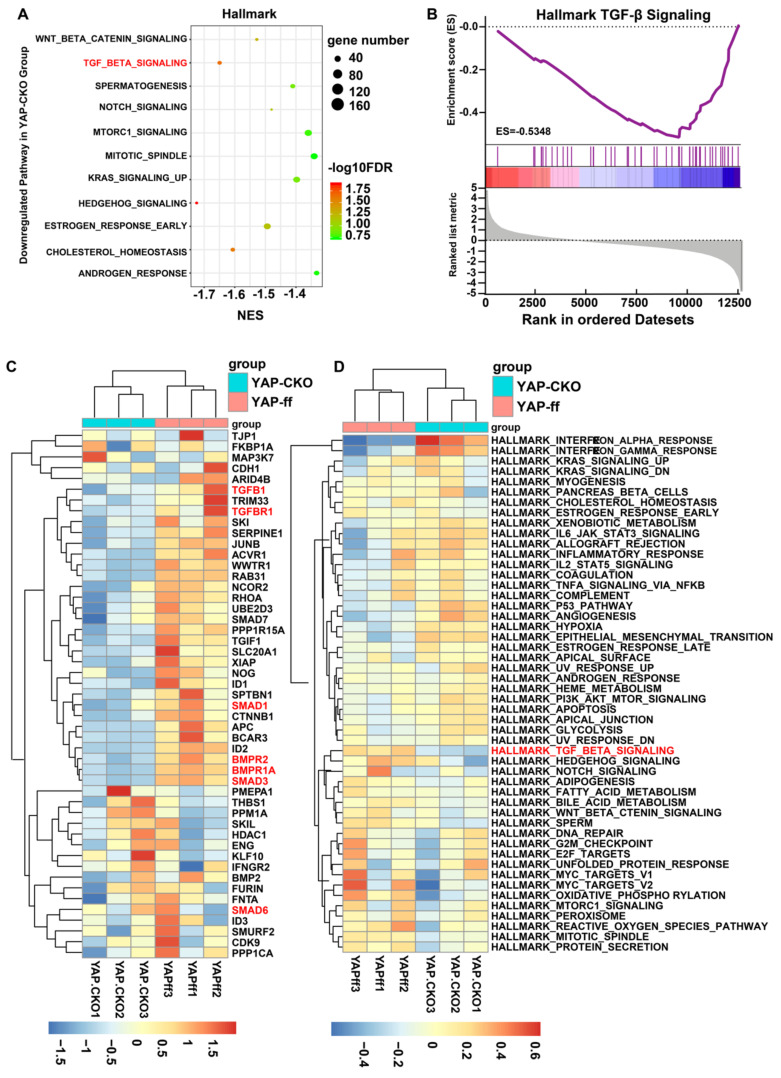
** GSEA and GSVA in YAP^+/+^ vs YAP^-/-^ astrocytes based on RNA-seq.** (A) The 11 down-regulated gene set with statistically significant (FDR < 0.25). (B) The enrichment plot of TGF-β signaling gene sets. (C) The clustering heatmap of 49 genes in TGF-β signaling gene sets. Red, blue and white respectively represent high expression level, low expression level and no expression difference among the genes. (D) GSVA-derived clustering heatmap of differentially expressed gene sets. Red, blue and white respectively represent high expression level, low expression level and no expression difference among the genes.

How does YAP regulate the neuroinflammation in MS-ON? To address this question, mRNA sequencing of YAP^+/+^ and YAP^-/-^ astrocytes was performed. GSEA was implemented with all expressed genes on account of hallmark gene set. The results showed that YAP was functionally related to Hedgehog signaling and TGF-β signaling (Figure 6A). Among these, TGF-β signaling has been reported to be highly associated with inflammation 30 and enrichment plot showed that TGF-β1 was markedly downregulated in YAP^-/-^ astrocytes, compared with that in YAP^+/+^ astrocytes (Figure 6B). To investigate each gene that was enriched in TGF-β signaling gene set, hierarchical clustering analysis was performed for 49 genes of YAP^+/+^ and YAP^-/-^ groups. The gene expression was evidently different in two groups by Tree View (Figure 6C). In addition, similar conclusions were obtained from GSVA (Figure 6D) and TGF-β signaling was common to top 3 gene sets of GSEA and GSVA both.

To confirm the results of bio-informatics analysis, qPCR analysis was performed in YAP^+/+^ and YAP^-/-^ astrocytes. The mRNA levels of TGF-β signaling, such as TGF-β1, Smad1 and Smad3 were markedly decreased in YAP^-/-^ astrocytes, compared with that in YAP^+/+^ astrocytes (Supplementary Figure S5A), which suggested YAP deletion in astrocytes might down-regulate TGF-β signaling pathway *in vitro.* Additionally, double-immunostaining of TGF-β1 and GFAP showed that the expression of astrocytic TGF-β1 was decreased in optic nerve of YAP^GFAP^-CKO EAE mice, compared with that in YAP^f/f^ EAE mice (Supplementary Figure S5B-C). To further observe the relationship between YAP and TGF-β signaling pathway *in vivo,* TGF-β signaling pathway, including TGF-β1, Smad1, Smad2, Smad3 and Smad4, was evaluated by qPCR and showed that TGF-β signaling was decreased in YAP^GFAP^-CKO EAE mice, compared with that in YAP^f/f^ EAE mice (Supplementary Figure S5D). These results suggest that YAP deletion in astrocytes may down-regulate TGF-β signaling pathway *in vitro* and* in vivo*, which may result into more severe inflammation in optic nerve of EAE mice.

### Activation of TGF-β signaling pathway partially relieves the neuroinflammation and demyelination in optic nerve and retina of YAP^GFAP^-CKO EAE mice

**Figure 7:**
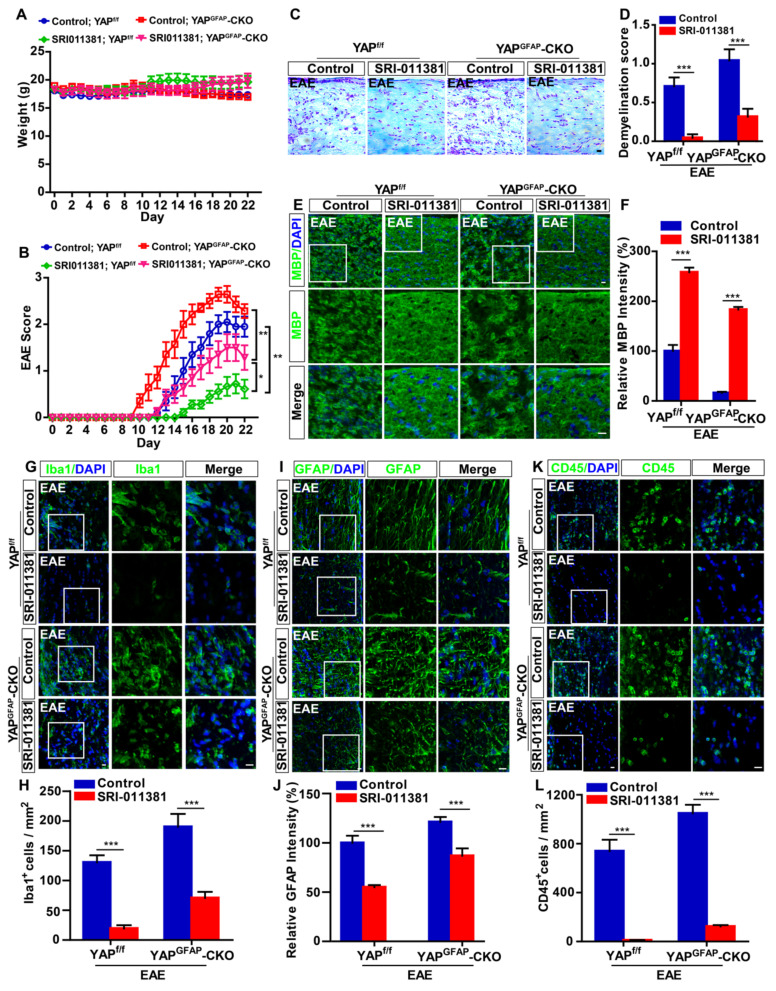
** Activation of TGF-β signaling reduces the inflammatory infiltration and demyelination in the optic nerve of EAE mice.** (A) The body weight of control-treated YAP^f/f^ (n = 10), control-treated YAP^GFAP^-CKO mice (n = 7), SRI-011381-treated YAP^f/f^ (n = 9) and SRI-011381-treated YAP^GFAP^-CKO mice (n = 8) from 0 to 22 dpi during the process of EAE modeling. (B) The EAE score of control-treated YAP^f/f^ mice (n = 10), control-treated YAP^GFAP^-CKO mice (n = 7), SRI-011381-treated YAP^f/f^ mice (n = 9) and SRI-011381-treated YAP^GFAP^-CKO mice (n = 8) 0 to 22 dpi during the process of EAE modeling. (C) LFB staining in the optic nerve of control-treated YAP^f/f^ EAE and YAP^GFAP^-CKO EAE mice, SRI-011381-treated YAP^f/f^ EAE and YAP^GFAP^-CKO EAE mice. (D) Quantitative analysis of the demyelination score as shown in (C) (n = 7 per group). (E, G, I, K) Immunostaining of MBP (green) (E), Iba1 (green) (G), GFAP (green) (I) or CD45 (green) (K) in the optic nerve of control-treated YAP^f/f^ EAE and YAP^GFAP^-CKO EAE mice, SRI-011381-treated YAP^f/f^ EAE and YAP^GFAP^-CKO EAE mice. (F, J) Quantitative analysis of the relative intensity of MBP (F) or GFAP (J) as shown in (E, I) (n = 6 per group, normalized to control). (H, L) Quantitative analysis of the density of Iba1^+^ cells (H) or CD45^+^ cells (L) as shown in (G, K) (n = 7 per group). Images of selected regions (white squares) were shown at higher magnification. Data were mean ± SEM, two-way ANOVA with Bonferroni's post-tests, compared with control group, ^*^P < 0.05, ^**^P < 0.01, ^***^P < 0.001. Scale bars, 20 μm.

To further investigate the relationship of YAP and TGF-β1 in optic neuritis, YAP^GFAP^-CKO mice and their littermate control YAP^f/f^ mice were performed with intraperitoneally injection of SRI-011381 (an agonist of TGF-β pathway) simultaneously after induction of EAE. As shown in Figure 7A-B, results revealed no significant differences in body weight between SRI-011381 treatment YAP^f/f^ and YAP^GFAP^-CKO mice during the process of EAE modeling (Figure 7A). However, SRI-011381 treatment significantly reduced the clinical scores in YAP^GFAP^-CKO EAE mice, compared with control-treated YAP^GFAP^-CKO EAE mice (Figure 7B). Moreover, HE and Nissl staining revealed that SRI-011381 treatment significantly inhibited inflammatory infiltration and relieved the loss of neurons in YAP^GFAP^-CKO EAE mice (Supplementary Figure S6A-B).

Additionally, SRI-011381 treatment relieved the demyelination in optic nerve of YAP^f/f^ EAE mice and YAP^GFAP^-CKO EAE mice, compared with that in control mice (Figure 7C-F). However, the demyelination degree of SRI-011381-treated YAP^GFAP^-CKO EAE mice was more severe than that of SRI-011381-treated YAP^f/f^ EAE mice (Figure 7 C-F). As shown in Figure 7G-L, immunostaining of Iba1, GFAP and CD45 also indicated that the obvious inflammatory infiltration was ameliorated in optic nerve of YAP^f/f^ EAE and YAP^GFAP^-CKO EAE mice treated with SRI-011381. Interestingly, immunostaining of Iba1 and GFAP in retina also suggested that the SRI-011381 treatment did suppress the inflammatory infiltration (Supplementary Figure S6C-F) and rescue RGCs loss (Supplementary Figure S6G-H) in retina of YAP^GFAP^-CKO EAE mice. Taken together, these results indicate that activation of TGF-β signaling pathway partially relieves the neuroinflammation and demyelination in optic nerve and retina of YAP^GFAP^-CKO EAE mice.

### Activation of YAP signaling relieves the neuroinflammatory infiltration and demyelination in optic nerve, and reduces loss of RGCs in retina of EAE mice

**Figure 8:**
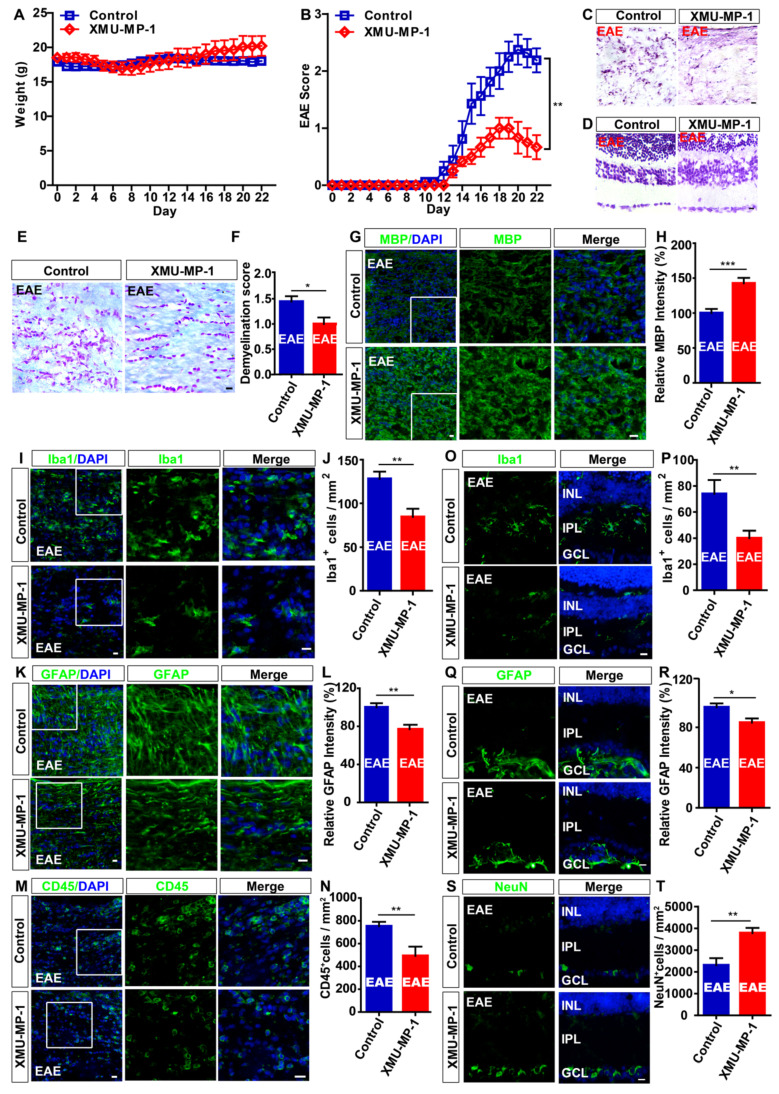
** Activation of YAP signaling by XMU-MP-1 partially reduces the inflammation and demyelination of optic nerve, and the loss of RGCs in EAE mice.** (A) The body weight of control (n = 8) and XMU-MP-1 (n = 6) treatment EAE mice from 0 to 22 dpi during EAE modeling (two-way ANOVA with Bonferroni's post-tests). (B) The EAE score of control (n = 8) and XMU-MP-1 (n = 6) treatment EAE mice 0 to 22 dpi during the process of EAE modeling (two-way ANOVA with Bonferroni's post-tests). (C) HE staining of optic nerve obtained from control and XMU-MP-1-treated EAE mice. (D) Nissl staining of retina obtained from control and XMU-MP-1-treated EAE mice. (E) LFB staining of optic nerve obtained from control and XMU-MP-1-treated EAE mice. (F) Quantitative analysis of the demyelination score of control and XMU-MP-1-treated EAE mice as shown in (E) (n = 7 per group, Student's t-test). (G, I, K, M) Immunostaining of MBP (green) (G), Iba1 (green) (I), GFAP (green) (K) or CD45 (green) (M) in the optic nerve of control and XMU-MP-1-treated EAE mice. (H, L) Quantitative analysis of the relative MBP intensity (H) or GFAP intensity (L) as shown in (G, K) (n = 7 per group, normalized to control, Student's t-test). (J, N) Quantitative analysis of the density of Iba1^+^ cells (J) or CD45^+^ cells (N) as shown in (I, M) (n = 6 per group, Student's t-test). (O, Q, S) Immunostaining of Iba1 (green) (O), GFAP (green) (Q) or NeuN (green) (S) in the retina of control and XMU-MP-1-treated EAE mice. (P, T) Quantitative analysis of the density of Iba1^+^ cells (P) or NeuN^+^ cells (T) as shown in (O, S) (n = 5 per group, Student's t-test). (R) Quantitative analysis of the relative GFAP intensity as shown in (Q) (n = 7 per group, normalized to control, Student's t-test). Images of selected regions (white squares) were shown at higher magnification. Data were mean ± SEM, compared with control group,^*^P < 0.05, ^**^P < 0.01, ^***^P < 0.001. Scale bars, 20 μm.

Recent studies showed that XMU-MP-1 can activate YAP by inhibiting MST1 and MST2 of Hippo signaling pathway, which is reported to promote regeneration in the liver and spinal cord injury (SCI) 43, 54, and protect the heart 55 and brain 56. As expected, immunohistochemistry and immunostaining showed that XMU-MP-1 treatment indeed significantly activated YAP in optic nerve astrocytes of mice (Supplementary Figure S7A-D). As shown in Figure 8A, there was no significant difference in the body weight between control and XMU-MP-1-treated mice during the process of EAE, however, XMU-MP-1 treatment significantly reduced the clinical scores (Figure 8B).

Then, the gross morphology of optic nerve and retina was observed by HE and Nissl staining and showed that XMU-MP-1 treatment significantly relieved the damage in optic nerve (Figure 8C-D). LFB staining and immunostaining of MBP showed that, the demyelination in optic nerve was relieved in the XMU-MP-1-treated EAE mice, compared with that in control-treated EAE mice (Figure 8E-H). As shown in Figure 8I-N, immunostaining of Iba1, GFAP and CD45 also indicated that XMU-MP-1 treatment did ameliorate the inflammatory infiltration in optic nerve. As expected, the inflammatory infiltration was significantly decreased in retina of XMU-MP-1-treated EAE mice (Figure 8O-R). Meanwhile, XMU-MP-1 treatment also reduced the loss of RGCs in retina of EAE mice (Figure 8S-T). Furthermore, results from qPCR showed that XMU-MP-1 treatment upregulated TGF-β signaling in optic nerve of EAE mice (Supplementary Figure S5E), which was consistent with the findings from our bio-informatics analysis. Taken together, these results indicated that activation of YAP signaling by XMU-MP-1 could reduce inflammatory infiltration and demyelination and the loss of RGCs in EAE mice through upregulating the TGF-β signaling.

## Discussion

**Figure 9:**
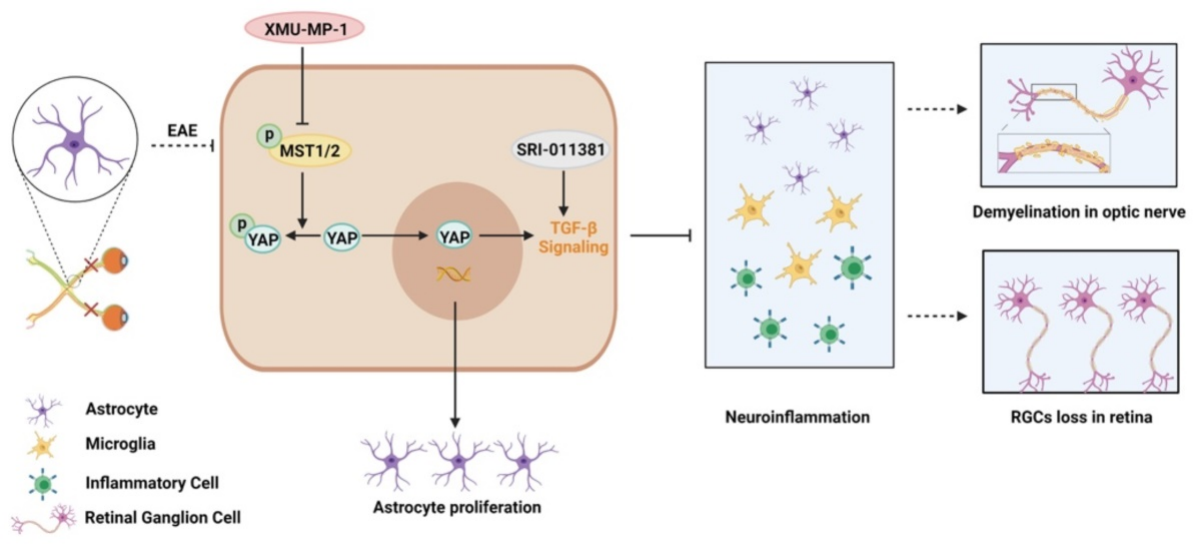
** Working model of YAP's functions in protection the optic nerve and retina through TGF-β signaling in an EAE model.** YAP signaling is activated in optic nerve astrocytes of EAE mice. Activation of YAP signaling promotes the proliferation of astrocytes, and upregulates TGF-β signaling to prevent the neuroinflammation in optic nerve and retina of EAE mice, which further inhibits the demyelination of optic nerve and the loss of RGCs in retina. XMU-MP-1 is an inhibitor of Hippo kinase MST1/2 to activate YAP signaling. SRI-011381 is an agonist of TGF-β signaling.

In this study, we provide evidence of astrocytic YAP's function in the neuroinflammation and demyelination of optic neuritis. YAP prevents the neuroinflammation and demyelination in optic nerve and retina of EAE mice through upregulation of TGF-β signaling pathway (Figure 9).

The characteristically pathological changes of EAE are multiple demyelinating plaques, reactive glial hyperplasia and axonal damage 57. The mammalian optic nerve contains three types of optic glial cells, including oligodendrocytes, microglia and astrocytes 51, 58. Previous researches have paid much more attention to microglia, immune cells and oligodendrocytes in EAE 59, 60. Nevertheless, recent accumulating evidences suggest that astrocytes are also key regulatory cells for CNS neuroinflammation in MS/EAE 12, 61. Reactive astrogliosis is a prominent feature of the chronic and widespread adaptive immune inflammation of CNS that occurs during conditions such as EAE and MS, and the basic process of reactive astrogliosis has long been regarded as primarily detrimental in MS and EAE 62, 63. Several studies have pointed out that perforin can be expressed by reactive astrocytes in MS, which represents the ongoing inflammation of nerves 64. Ablation of proliferating reactive astrocytes increased the spread of infiltrating inflammatory cells and the exacerbation of EAE clinical signs 13. Consistent with previous studies, our results in this study revealed that astrocytes were activated in optic nerve of EAE (Supplementary Figure S1). But the mechanism underlying in MS-ON that regulated by astrocytes remains unclear.

YAP acts as a transcriptional co-activator and plays an important role in CNS 65. Our previous studies have shown that YAP is upregulated and activated in GFAP^+^ astrocytes after SCI 43. Consistent with our previous studies, we also found that YAP was upregulated and activated in optic nerve astrocytes of EAE mice (Figure 1). However, interestingly, our histological observations revealed that YAP knockout in astrocytes did not affect the visual development (Supplementary Figure S3). Previous studies have shown that deletion of YAP and TAZ may affect development of optic nerve and retina. For example, YAP-deficient retinal progenitors displayed decreased S-phase cells and altered cell cycle progression in the developing mouse eye 66; activated YAP/TAZ promotes rescuing angiogenesis and barrier genesis in retina 67. It's probably because GFAP^+^ astrocytes are late to emerge during the embryonic development of optic nerve, and there is virtually no GFAP^+^ astrocytes at E16, and only 8% of the purified cells are GFAP^+^ astrocytes at E17 68. Furthermore, astrocytes in optic nerve are actually not activated under physiological conditions 68, 69. However, in optic neuritis, the deletion of YAP in astrocytes aggravated the demyelination (Figure 2) and inflammatory infiltration (Figure 3) in optic nerve, promoted the activation of astrocytes and microglia, and induced a drastic increase of RGCs death in retina (Figure 5). The microglia may be activated by some factors secreted by YAP-deleted astrocytes. Studies have shown that the activation of microglia and the neuroinflammation may be excessive effects of the JAK-STAT inflammatory pathway through YAP-deficient astrocytes 21. In addition, abnormal activation of astrocytes has been associated with upregulation and secretion of cytokines, such as TNF-α and IL-3, as well as chemokines like Ccl3, Ccl4 and Ccl8, which may also attract microglia and increase neuroinflammation 70-72. Consistent with previous study, we found that increase of Ccl8, Ccl9, TNF-α and IL-1β from YAP-deleted astrocytes might promote activation of microglia and neuroinflammation (Supplementary Figure S4). Taken together, these results indicate that conditional YAP knockout in astrocytes exacerbates inflammatory infiltration in the optic nerve of EAE mice.

How does YAP signaling in astrocytes suppress the neuroinflammation of MS-ON? Astrocytic TGF-β signaling plays multiple functions in protecting against chronic and widespread adaptive immune inflammation of the CNS. For example, inhibiting astrocytic TGF-β signaling was found to aggravate inflammation in the peri-infarct cortex during the subacute period following distal middle cerebral occlusion 73, and astrocytic TGF-β signaling limits inflammation and reduces neuronal damage during CNS toxoplasma infection 74. Additionally, astrocytic TGF-β1 protects synapses against Aβ oligomers in Alzheimer's disease model 75. Consistent with these previous studies, we found that downregulation of TGF-β signaling in YAP^GFAP^-CKO EAE mice promoted neuroinflammation. These results suggest that astrocytic TGF-β signaling has conservative anti-neuroinflammation roles in different disease model. Furthermore, numerous studies have demonstrated that TGF-β is significantly upregulated in MS patients, with its injection into EAE mice models associated with a reduction in frequency and severity of attacks 31-33. Conversely, lacking TGF-β was found to develop a multifocal inflammatory disease 76, indicating that TGF-β might be playing an important role in MS or EAE. Previous studies have shown that YAP can regulate TGF-β signaling in various situations 35, 77. For example, increased nuclear YAP/TAZ overcomes TGF-β1-mediated tumor suppressive functions (e.g. cytostasis) and concomitantly drives tumorigenic transcriptional events by promoting the activity of YAP/TEAD-SMAD complexes in breast cancer cells 78. In skin wound healing, the knockdown of YAP /TAZ markedly delayed the rate of wound closure and reduced the expression of TGF-β1, and YAP/TAZ also modulated the expression of TGF-β signaling pathway components such as Smad2, Smad7 and p21 79. Consistent with these previous studies, our RNA-seq results showed that TGF-β1 related signaling, including TGF-βR1, smad1, smad3, BMPR1 and BPMR2 were significantly decreased in YAP^-/-^ groups (Figure 6). To strengthen the links between YAP and TGF-β signaling, TGF-β related signaling pathways in mice, either competent or lacking YAP, administered with XMU-MP-1, was evaluated by qPCR, including TGF-β1, Smad1, Smad2, Smad3 and Smad4 (Supplementary Figure S5), and the results of qPCR further confirmed the sequencing analysis. Therefore, YAP might act as a positive up-stream regulator of TGF-β1/Smad signaling, leading to protect the optic nerve and retina against damage in EAE mice. But our previous study showed that TGF-β1 was upregulated in astrocytes following YAP deletion 21. This may be due to different model and different assays (PCR array VS RNA-seq). The present study had other evidences to support the hypothesis that astrocytic YAP suppresses neuroinflammation in optic neuritis by upregulating TGF-β1 signaling. For example, SRI-011381 (an agonist of TGF-β pathway) treatment partially rescued infiltration of CD45^+^ cells and activated microglia in YAP^GFAP^-CKO EAE mice, compared with that in control mice. Although we could not exclude the possibility that SRI-011381 affected other cells in EAE mice, we found that TGF-β signaling was downregulated by YAP knockout in astrocytes based on RNA-seq, qPCR and immunostaining (Figure 6 and Supplementary Figure S5). These results suggest that TGF-β signaling may be a downstream of YAP signaling in regulation of neuroinflammation in EAE. However, detailed mechanism how YAP regulates expression of TGF-β1 in EAE requires further investigation.

To further elucidate YAP's therapeutic effect on recovery of optic neuritis, we treated mice with XMU-MP-1 to activate YAP signaling after EAE induction. Results showed that treatment relieved inflammatory infiltration and demyelination in optic nerve after EAE induction, consistent with our previous study which also has shown that XMU-MP-1 exerts a certain degree of therapeutic effect on SCI 43. Previous studies have shown that YAP is mainly expressed in astrocytes, but not in microglia and T cells 21, 43. Consistent with these previous studies, results of the present study showed that YAP was mainly expressed in astrocytes, and not microglial cells (Figure 1). Therefore, XMU-MP-1 treatment mainly affects astrocytes, whereas the responses of CD45^+^ cells or microglia to XMU-MP-1 treatment may be indirect. Taken together, these results suggest that YAP signaling could be a new therapeutic target for treatment of MS-ON.

## Conclusions

In summary, these results suggest that YAP may upregulate TGF-β signaling pathway to prevent inflammatory infiltration, demyelination and RGCs death in optic neuritis. These results not only reveal the unrecognized function of YAP signaling in optic neuritis, but also further link YAP with TGF-β1, which may provide a new potential therapeutic target for optic neuritis in clinic by selectively enhancing the function of YAP-TGF-β signaling pathway.

## Supplementary Material

Supplementary figures.

## Abbreviations

BSA: bovine serum albumin
CKO: Conditional knockout
CNS: central nervous system
dpi: day post injection
EAE: experimental autoimmune encephalomyelitis
EM: electron microscopy
GSEA: gene set enrichment analysis
GSVA: gene set variation analysis
HE: Hematoxylin-Eosin
LFB: Luxol Fast Blue
MBP: myelin basic protein
MOG_35-55_: myelin oligodendrocyte glycoprotein 35-55
MS: multiple sclerosis
MS-ON: MS related optic neuritis
PBS: phosphate-buffered saline
PFA: paraformaldehyde
qPCR: quantitative real-time PCR
RGCs: retinal ganglion cells
SCI: spinal cord injury
TAZ: transcriptional coactivator with PDZ-binding motif
TGF-β: transforming growth factor-β
YAP: yes-associated protein
