# Astrocytic YAP protects the optic nerve and retina in an experimental autoimmune encephalomyelitis model through TGF-β signaling: Erratum

**DOI:** 10.7150/thno.100784

**Published:** 2025-02-15

**Authors:** Qian Wu, Xuemeng Miao, Jingjing Zhang, Ludan Xiang, Xiuchun Li, Xiaomei Bao, Siyu Du, Mianxian Wang, Shuangda Miao, Yiren Fan, Wei Wang, Xingxing Xu, Xiya Shen, Danlu Yang, Xiwu Wang, Yuanyuan Fang, Lixin Hu, Xuyi Pan, Haoru Dong, Hui Wang, Ying Wang, Jia Li, Zhihui Huang

**Affiliations:** 1 School of pharmacy and Holistic Integrative Pharmacy Institutes, Hangzhou Normal University, Hangzhou, Zhejiang, 311121, China.; 2 School of Mental Health, Wenzhou Medical University, Wenzhou, Zhejiang, 325035, China.; 3 Phase I Clinical Research Center, Zhejiang Provincial People's Hospital of Hangzhou Medical College, Hangzhou, Zhejiang, 310053, China.; 4 The First Affiliated Hospital of Wenzhou Medical University, Wenzhou, Zhejiang, 325035, China.; 5 School of Basic Medical Sciences, Wenzhou Medical University, Wenzhou, Zhejiang, 325035, China.

The authors apologize that the original version of the above article contains errors that need to be corrected. An incorrect image for XMU-MP-1 group in Supplemental Data Figure S7A was inserted during Figure S7A organization. The authors have corrected the typical images of Supplemental Data Figure S7A and declare that these corrections do not change the results or conclusions of their paper. The authors sincerely apologize to the Journal and its readers for the confusion this may have caused. The corrected version of Supplemental Data Figure S7A appears below.

## Figures and Tables

**Figure A:**
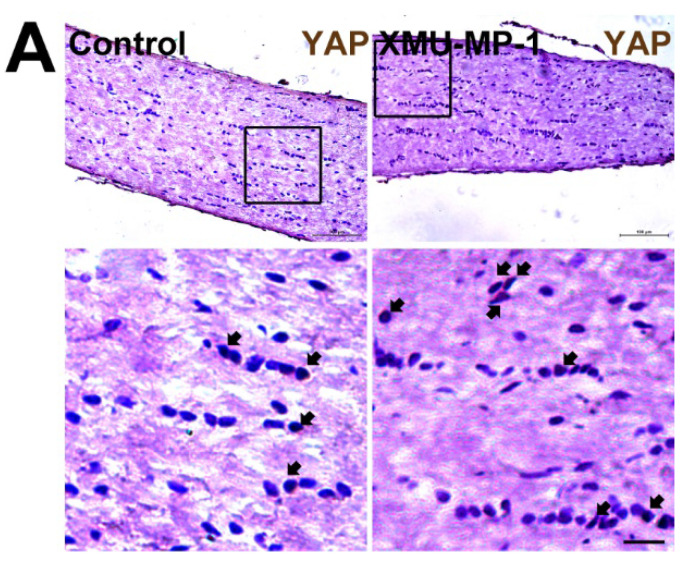
Corrected figure for original Supplemental Data Figure S7A.

