# Supplementary material for: Astrocytic YAP protects the optic nerve and retina in an experimental autoimmune encephalomyelitis model through TGF-β signaling
Source: Theranostics. 2021 Jul 25;11(17):8480–99. doi: 10.7150/thno.60031 (PMC8344002; doi:10.7150/thno.60031)
Supplement: Supplementary file 1 — Supplementary figures. [file thnov11p8480s1.pdf]

Supplemental data

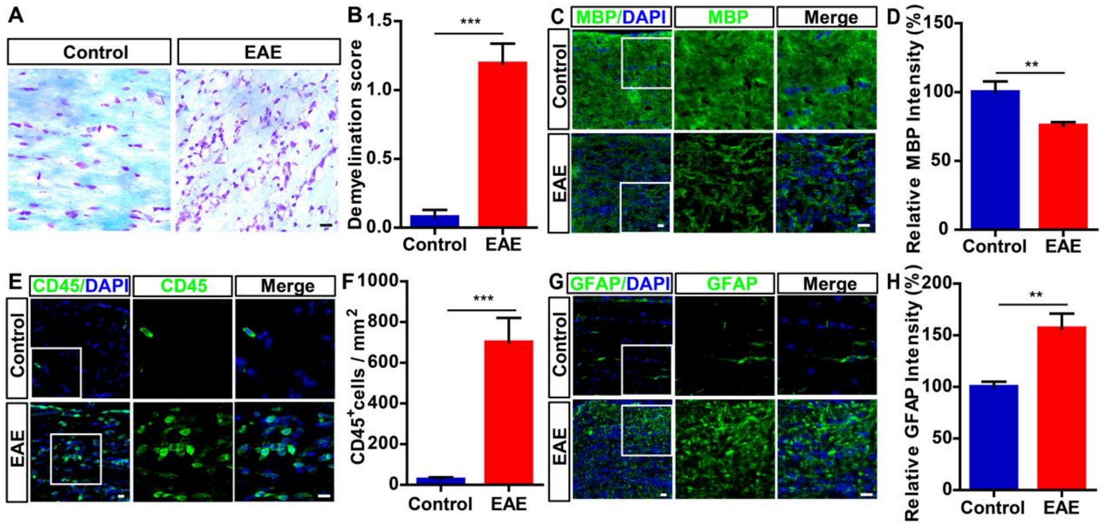

Figure S1. Establishment of EAE mice model. (A) LFB staining in the optic nerve of control and EAE mice. (B) Quantitative analysis of the demyelination score of control and EAE mice as shown in (A) (n = 7 per group). (C, E, G) Immunostaining of MBP (green) (C), CD45 (green) (E) or GFAP (green) (G) in the optic nerve of control and EAE mice. (D, H) Quantitative analysis of the relative intensity of MBP (D) or GFAP (H) as shown in (C, G) (n = 7 per group, normalized to control group). (F) Quantitative analysis of the density of $CD45^+$ cells as shown in (E) (n = 7 per group). Images of selected regions (white squares) were shown at higher magnification and the right panels were enlarged. Data were mean ± SEM, Student's t-test, compared with control group, $^{**}P < 0.01$, $^{***}P < 0.001$. Scale bars, 20 μm.

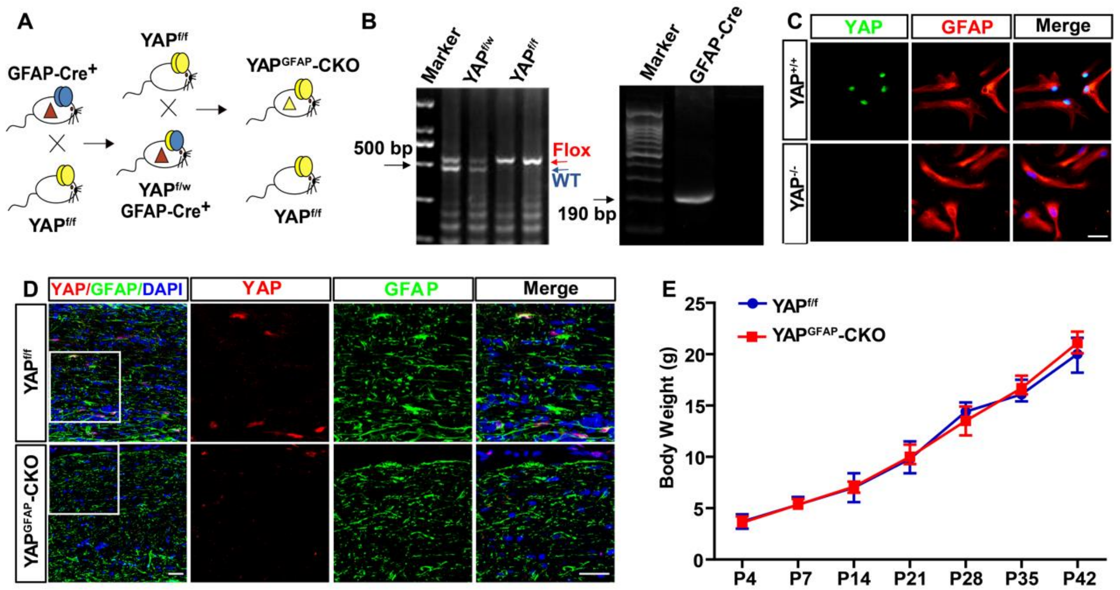

Figure S2. Identification of YAP^{GFAP}-CKO mice. (A) The flow chart showed the process of obtaining YAP^{GFAP}-CKO mice and their littermate control mice. (B) Genotyping was detected by agarose gel electrophoresis. (C) Double immunostaining of YAP (green) and GFAP (red) in cultured YAP$^{+/+}$ and YAP$^{-/-}$ astrocytes. (D) Double immunostaining of YAP (red) and GFAP (green) of optic nerve obtained from 2-month YAP$^{f/f}$ and YAP^{GFAP}-CKO mice. (E) Body weight of YAP$^{f/f}$ and YAP^{GFAP}-CKO mice at different developmental stages (n = 10 per group). Data were mean ± SEM, two-way ANOVA with Bonferroni's post-tests, compared with YAP$^{f/f}$ group. Scale bars, 20 μm.

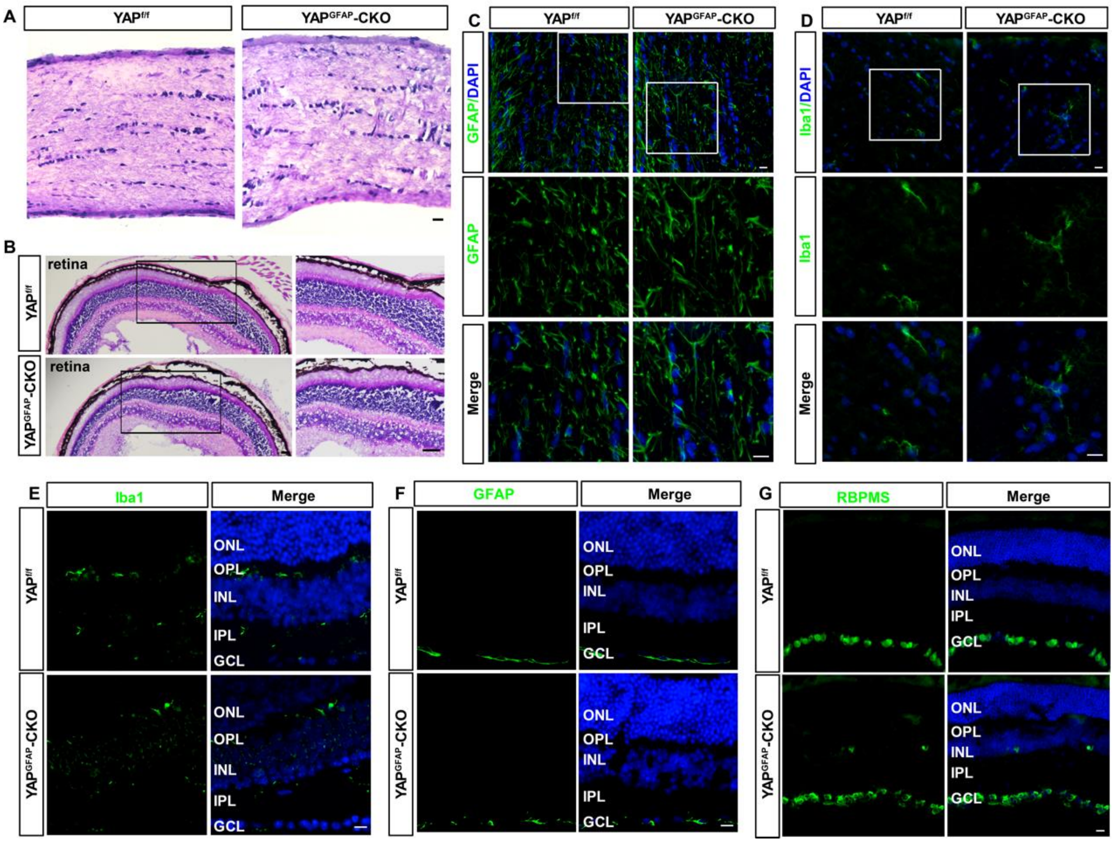

Figure S3. Normal development of optic nerve and retina in YAP^{GFAP}-CKO mice. (A-B) HE staining of optic nerve (A) or retina (B) obtained from 2-month YAP$^{f/f}$ and YAP^{GFAP}-CKO mice. (C-D) Immunostaining of GFAP (green) (C) or Iba1 (green) (D) in the optic nerve of 2-month YAP$^{f/f}$ and YAP^{GFAP}-CKO mice. (E-G) Immunostaining of Iba1 (green) (E), GFAP (green) (F) or RBPMS (green) (G) in the retina of 2-month YAP$^{f/f}$ and YAP^{GFAP}-CKO mice. Images of selected regions (white squares) were shown at higher magnification. Scale bars, 20 μm.

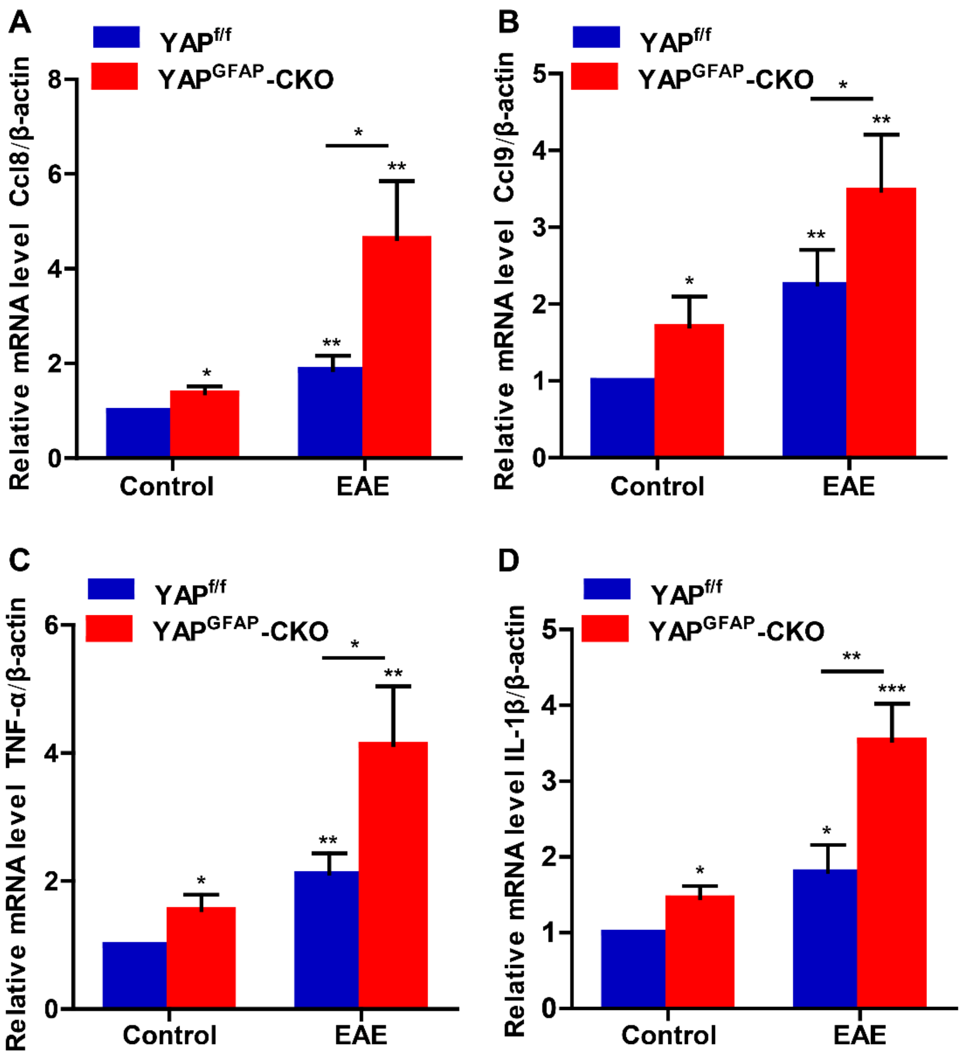

Figure S4. Expression levels of some cytokines and chemokines in the optic nerve of YAP$^{f/f}$ and YAP^{GFAP}-CKO mice, YAP$^{f/f}$ EAE and YAP^{GFAP}-CKO EAE mice. (A-D) qPCR analysis of the relative mRNA levels of Ccl8 (A), Ccl9 (B), TNF-α (C) and IL-1β (D) in the optic nerve of YAP$^{f/f}$ and YAP^{GFAP}-CKO mice, YAP$^{f/f}$ EAE and YAP^{GFAP}-CKO EAE mice (normalized to control group, n = 8 per group). Data were mean ± SEM, two-way ANOVA with Bonferroni's post-tests, compared with YAP$^{f/f}$ group. $^{*}P < 0.05$, $^{**}P < 0.01$, $^{***}P < 0.001$.

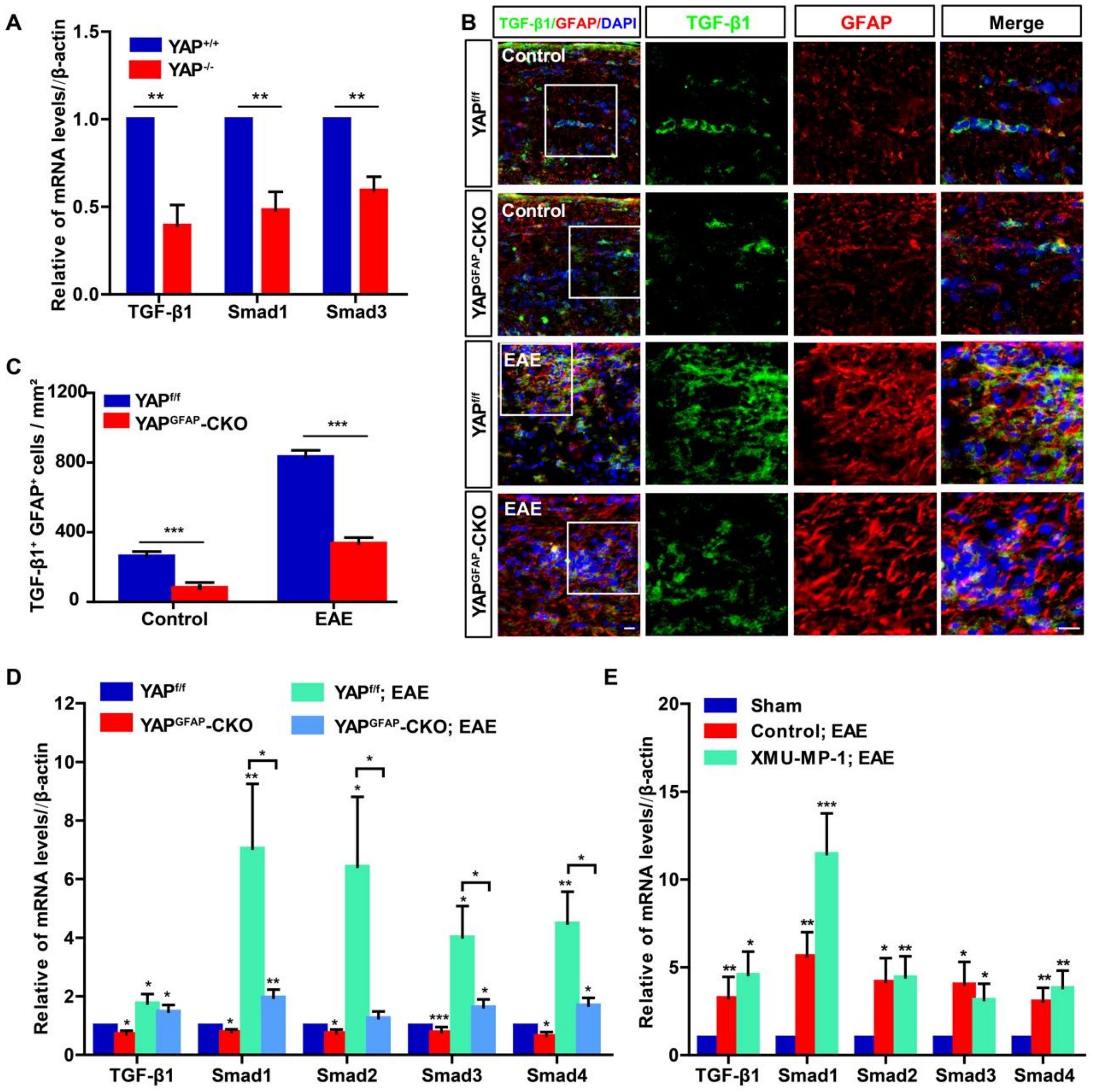

Figure S5. Analysis of TGF-β signaling under various conditions by qPCR and immunostaining. (A) qPCR analysis of the relative mRNA level of TGF-β1, Smad1 and Smad3 in cultured YAP$^{+/+}$ and YAP$^{-/-}$ astrocytes (n = 8 per group, normalized to YAP$^{-/-}$ group, compared with YAP$^{-/-}$ group). (B) Double immunostaining of TGF-β1 (green) and GFAP (red) in the optic nerve of YAP$^{f/f}$ and YAP^{GFAP}-CKO mice, YAP$^{f/f}$ EAE and YAP^{GFAP}-CKO EAE mice. (C) Quantitative analysis of the density of TGF-β1^{+} astrocytes as shown in (B) (n = 7 per group, compared with YAP$^{f/f}$ group). Images of selected regions (white squares) were shown at higher magnification. (D) qPCR analysis of the relative mRNA levels of TGF-β1, Smad1, Smad2, Smad3 and Smad4 in the optic nerve of YAP$^{f/f}$ and YAP^{GFAP}-CKO mice, YAP$^{f/f}$ EAE and YAP^{GFAP}-CKO EAE mice (n = 10 per group, normalized to control group, compared with YAP$^{f/f}$ group). (E) qPCR analysis of the relative mRNA levels of TGF-β1, Smad1, Smad2, Smad3 and Smad4 in the optic nerve of sham, control-treated and XMU-MP-1-treated EAE mice (n = 7 per group, normalized to sham group, compared with sham group). Data were

mean ± SEM, two-way ANOVA with Bonferroni's post-tests, $^{*}P < 0.05$, $^{**}P < 0.01$, $^{***}P < 0.001$. Scale bars, 20 μm.

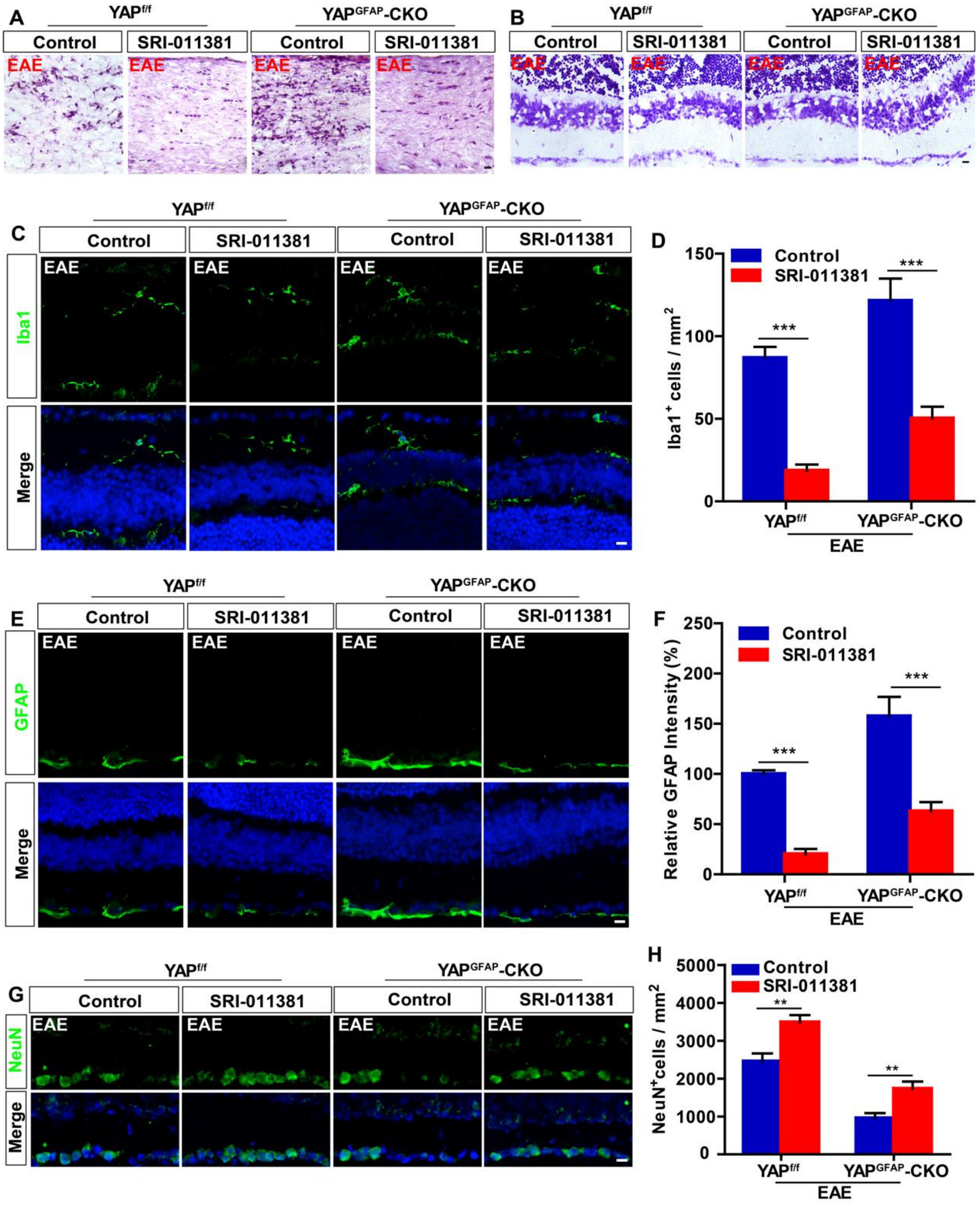

Figure S6. Activation of TGF-β signaling reduced the inflammatory infiltration and demyelination in optic nerve and retina of EAE mice. (A) HE staining of optic nerve obtained from control-treated YAP$^{f/f}$ EAE and YAP^{GFAP}-CKO EAE mice, SRI-011381-treated YAP$^{f/f}$ EAE and YAP^{GFAP}-CKO EAE mice. (B) Nissl staining of retina obtained from control-treated YAP$^{f/f}$ EAE and YAP^{GFAP}-CKO EAE mice, SRI-011381-treated YAP$^{f/f}$ EAE and YAP^{GFAP}-CKO EAE mice. (C, E, G) Immunostaining of Iba1 (green) (C), GFAP (green) (E) or NeuN (green) (G) in the retina of control-treated YAP$^{f/f}$ EAE and YAP^{GFAP}-CKO EAE mice, SRI-011381-treated YAP$^{f/f}$ EAE and YAP^{GFAP}-CKO EAE mice. (D, H) Quantitative analysis of the density of Iba1^{+} cells (D) or NeuN^{+} cells (H) as shown in (C, G) (n = 6 per group). (F) Quantitative analysis of

GFAP intensity as shown in (E) (n = 7 per group, normalized to $YAP^{f/f}$ group). Data were mean ± SEM, two-way ANOVA with Bonferroni's post-tests, compared with control group, $^{**}P < 0.01$, $^{***}P < 0.001$. Scale bars, 20 μm.

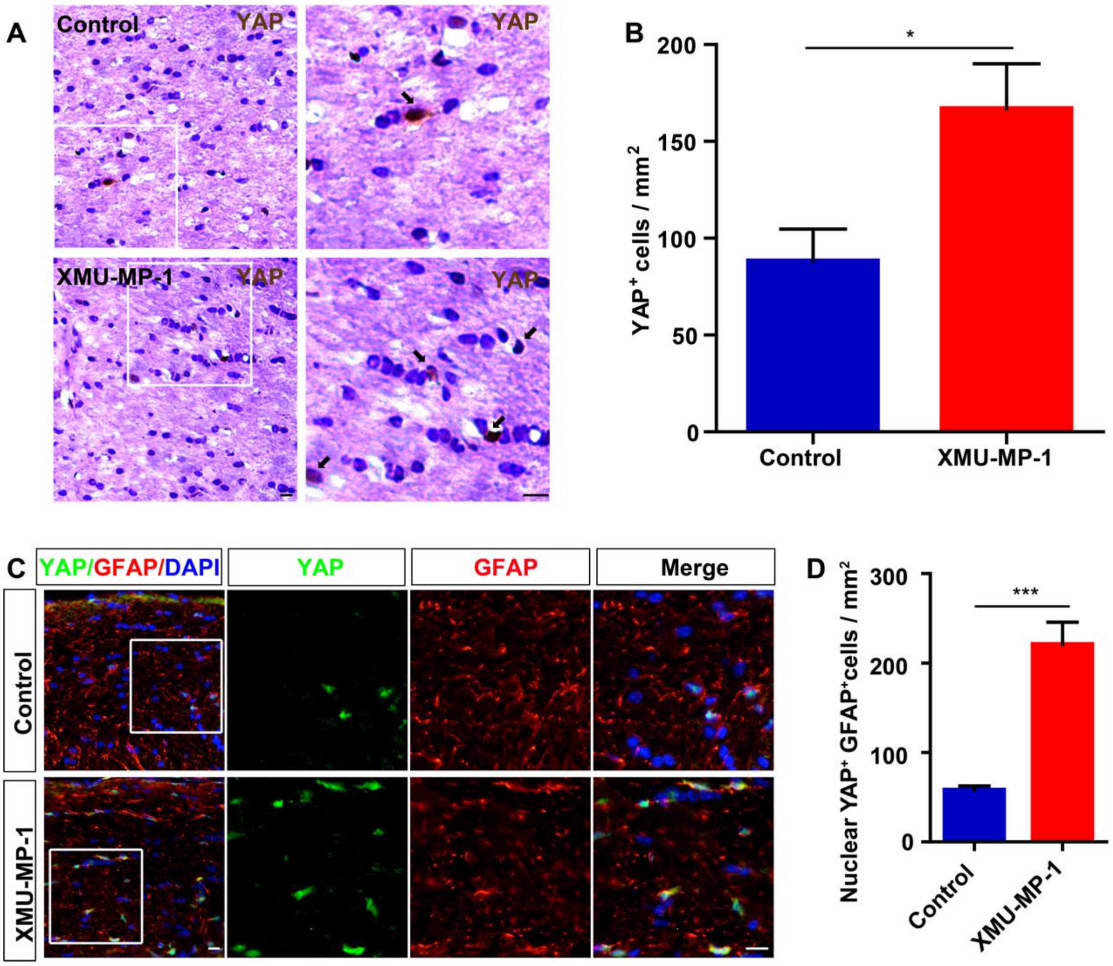

Figure S7. Activation of YAP in astrocytes of optic nerve by XMU-MP-1. (A) Immunohistochemistry of YAP in the optic nerve of control and XMU-MP-1-treated 2-month mice. (B) Quantitative analysis of the density of YAP^+ cells as shown in (A) (n = 6 per group). (C) Double immunostaining of YAP (green) and GFAP (red) in the optic nerve of control and XMU-MP-1-treated 2-month mice. (D) Quantitative analysis of the density of nuclear YAP^+ astrocytes as shown in (C) (n = 7 per group). Images of selected regions (white squares) were shown at higher magnification. Data were mean ± SEM, Student's t-test, compared with control mice, $^*P < 0.05$, $^{***}P < 0.001$. Scale bars, 20 μm.
